# The clinical and radiographic characteristics of avascular necrosis after pediatric femoral neck fracture: a systematic review and retrospective study of 115 patients

**DOI:** 10.1186/s13018-020-02037-2

**Published:** 2020-11-11

**Authors:** Pengfei Xin, Yonggang Tu, Zhinan Hong, Fan Yang, Fengxiang Pang, Qiushi Wei, Wei He, Ziqi Li

**Affiliations:** 1 The First Clinical Medical School, Guangzhou University of Chinese Medicine, Jichang Road 12#, District Baiyun, Guangzhou, Guangdong China; 2 Laboratory of Orthopaedics & Traumatology, Lingnan Medical Research Center, Guangzhou University of Chinese Medicine, Guangzhou, China; 3 Department of Orthopaedics, Dongguan Eastern Central Hospital, Dongguan, Guangdong China; 4 Department of Joint Surgery, The Third Affiliated Hospital of Guangzhou University of Chinese Medicine, Guangzhou, 510405 China

**Keywords:** Avascular necrosis, Femoral neck fractures, Child, Adolescent

## Abstract

**Background:**

Avascular necrosis (AVN) after pediatric femoral neck fracture (PFNF) showed poor prognosis, but its clinical and radiographic characteristics remained unclear.

**Methods:**

A systematic review and a retrospective study were performed to evaluate the clinical and radiographic characteristics of patients with AVN after PFNF.

**Results:**

A total of 686 patients with PFNF and 203 patients with AVN from 21 articles were analyzed. Ratliff’s classification was used in 178 patients, with types I, II, and III AVN accounting for 58.4%, 25.3%, and 16.3%, respectively. Ratliff’s assessment was used in 147 patients, of whom 88.4% had an unsatisfactory prognosis. In retrospective study, 115 patients with a mean age of 13.6 ± 2.0 years were included. The mean interval between AVN and PFNF was 13.7 ± 9.5 months. At the time of diagnosis, 59.1% cases were symptomatic and 65.2% progressed to collapsed stage. Fifty (43.5%), 61 (53.0%), and 4 patients (3.5%) were defined as types I, II, and III , respectively, via Ratliff’s classification. Thirteen (11.3%), 40 (34.8%), and 62 patients (53.9%) showed types A/B, C1, and C2 disease, respectively, via the JIC classification. Multivariate analysis demonstrated a strong relation between collapsed stage and symptomatic cases (OR = 6.25, 95% CI = 2.39–16.36) and JIC classification (OR = 3.41, 95% CI = 1.62–7.17).

**Conclusion:**

AVN after PFNF showed a tendency toward extensive necrotic lesions, presumably resulting in a rapid progression of femoral head collapse. And the symptoms and the JIC classification are other two risk factors of collapse progression.

**Supplementary Information:**

The online version contains supplementary material available at 10.1186/s13018-020-02037-2.

## Introduction

Pediatric femoral neck fractures (PFNFs) are rare but devastating injuries that are mostly induced by high-energy trauma in children and adolescents, with an incidence of less than 1% [1, 2]. Avascular necrosis (AVN) is the most common complication that occurs after PFNF, resulting in poor prognosis that is debilitating and potentially disabling in young populations. Accumulating evidence-based medical research has confirmed the high incidence of AVN after PFNF. For example, an average incidence of 23.5% was reported by a meta-analysis that included 30 studies and 935 patients in 2013 [3]. A similar outcome, an incidence of 24.5%, was confirmed repeatedly in a review of 239 cases of PFNF in 2019 [4]. However, the clinical and radiographic characteristics of AVN after PFNF remain foreign to most orthopedic surgeons because of the rare incidence of primary injury.

To the best of our knowledge, a handful of studies have specifically described the characteristics of AVN after PFNF. In 1962, Ratliff et al. [2] first described three patterns of AVN after PFNF according to a review of 29 cases: the highest incidence was of AVN occupying the total head (type I, 15 cases), followed by partial necrosis of the epiphysis (type II, 7 cases) and necrosis between the epiphyseal plate and the fracture line (type III, 7 cases). Numerous subsequent studies adapted this criterion (Table 1); however, the limited sample size of enrolled patients was insufficient for demonstrating the prognostic value of Ratliff’s classification. In addition, few studies have confirmed the relationship between the prognosis of AVN after PFNF and other recognized prognostic factors for most types of AVN, including hip symptoms, the presence of collapse at diagnosis, and the location of the lesion [25, 26].

**Table 1:** The outcomes of AVN after PFNF in the published literature as described by the Ratliff classification system and assessment

Authors	Year	Patients	Mean age, years (range)	AVN	Ratliff classification^*^			Ratliff assessment^**^	
					I	II	III	Satisfied	Unsatisfied
Stone et al. [5]	2015	22	11.0 (4.5–17.4)	8	5	2	1	3	5
Panigrahi et al. [6]	2015	28	10.5 (4–15)	4	0	2	2	NA	NA
Bukva et al. [7]	2015	28	10.7 (4–14)	11	6	3	2	NA	NA
Hadju et al. [8]	2011	8	11.6 (3–15)	1	0	1	0	NA	NA
Bali et al. [9]	2011	36	10.0 (3–16)	7	6	1	0	0	7
Nayeemuddin et al. [10]	2009	14	10.0 (6–14)	1	1	0	0	0	1
Inan et al. [11]	2009	39	11.1 (4–16)	11	8	1	2	1	10
Varshney et al. [12]	2009	21	11.8 (5–15)	3	1	2	0	NA	NA
Dhammi et al. [13]	2005	26	10.8 (3–17)	4	NA	NA	NA	0	4
Togrul et al. [14]	2005	61	10.2 (2–14)	9	8	1	0	NA	NA
Flynn et al. [15]	2002	18	8.0 (2–13)	1	0	1	0	NA	NA
Bagatur et al. [16]	2002	17	11.0 (7–14)	9	4	2	3	0	9
Mirdad et al. [17]	2002	14	9.1 (4–16)	7	4	3	0	NA	NA
Morsy et al. [18]	2001	53	10.2 (3–16)	21	NA	NA	NA	0	21
Ng et al. [19]	1996	32	9.5 (NA)	9	7	1	1	NA	NA
Forlin et al. [20]	1992	16	11.7 (4.6–16)	14	5	5	4	2	12
Canale et al. [21]	1977	60	9.7 (0.5–17)	26	21	1	4	1	25
Chong et al. [22]	1975	20	NA (5–19)	10	5	5	0	2	8
Zolczer et al. [23]	1972	27	NA (13–19)	7	2	5	0	5	2
Lam et al. [24]	1971	75	NA (≦ 17)	11	6	2	3	NA	NA
Ratliff [2]	1962	71	NA (< 17)	29	15	7	7	3	26
Total		686		203	104	45	29	17	130
Percentage				29.6%	58.4%	25.3%	16.3%	11.6%	88.4%

*AVN* avascular necrosis, *NA* missing value/not clear

*Classification of avascular necrosis as proposed by Ratliff

**Classification of final result according to Ratliff

As we believe, a specific description of the clinical and radiographic characteristics of AVN after PFNF is beneficial for understanding the potential disease progression and managing targeted treatments. Considering the low incidence of PFNF, we aimed to elucidate the clinical and radiographic characteristics of AVN after PFNF via (1) a systematic review of the literature since 1962 and (2) a retrospective cross-sectional study based on the clinical and radiographic data from a single center, with a hypothesis that AVN after PFNF might be a rapidly progressing disease with a high risk of femoral head collapse, very likely resulting from its tendency to extensively involve necrotic lesions.

## Methods

### Systematic review

We searched PubMed, Embase, and Web of Science databases with a computer. The literature on osteonecrosis after fracture of the femoral neck in pediatric populations published from January 1960 to November 2019 was comprehensively searched, and the following key words were used: “adolescent” and “teen” and “teenager” and “youths” as well as “femur neck fracture”, “femoral neck fracture”, “avascular necrosis of the femoral head”, “ischemic necrosis of the femoral head”, “aseptic necrosis of the femoral head”, and “femoral head necrosis”, etc. The language was limited to English (see Additional file 1 for the details of the search). In addition, we searched the missing documents from the references, which were retrieved by hand.

### Selection criteria

The inclusion criteria were as follows: (1) age was less than 19 years; (2) a definite history of femoral neck fractures was confirmed by imaging; (3) complications including avascular necrosis were described; and (4) the Ratliff classification was used to assess the degree of avascular necrosis, or the prognosis of patients with avascular necrosis was assessed by the Ratliff criteria [2], and the corresponding data were recorded in detail. The exclusion criteria were as follows: (1) AVN after femoral neck fracture was excluded in adults; (2) literature with incomplete Ratliff classification and prognostic data was excluded; and (3) single case reports and reviews were excluded.

### Literature screening and data extraction

The two authors (Pengfei Xin and Ziqi Li) independently evaluated the retrieved articles by reading the title and abstract and evaluated all the articles that might have met the requirements by obtaining the full text. Any differences between the two authors were settled through discussion. The data extracted from the articles that met the requirements included the following: the total number of patients, age of patients, number of patients with avascular necrosis, degree of avascular necrosis, and final prognosis of patients with avascular necrosis.

The degree of necrosis of the femoral head was assessed using the Ratliff classification: type I—diffuse increases in density of the femoral head accompanied by complete collapse of the epiphysis; type II—partial head involvement with accompanying slight epiphyseal collapse and osteonecrosis; and type III—areas of avascular necrosis, with the range of necrosis usually limited to between the epiphyseal and fracture lines. The data regarding types I, II, and III necrosis were extracted retrospectively. Ratliff’s assessment was used to evaluate the prognosis of osteonecrosis patients from both imaging and clinical aspects. The score of good indicated a satisfactory prognostic effect, while a score of poor indicated an unsatisfactory prognostic effect (Table 2). The data regarding satisfactory and unsatisfactory prognoses were extracted retrospectively.

**Table 2:** Classification and prognostic assessment system of avascular necrosis

Types	The evaluation index
Ratliff’s classification of avascular necrosis (AVN)	
Type I	Diffuse density increases in the femoral head accompanied by complete collapse of the epiphysis
Type II	Partial head involvement with slight accompanying epiphyseal collapse and osteonecrosis
Type III	Areas of avascular necrosis, with the range of necrosis usually limited to between the epiphyseal and fracture lines
Ratliff system of clinical and radiographic assessment	
Good	Clinical: no pain, normal or slightly limited hip movement, normal daily activity
	Radiographic: normal or mild deformity of the femoral neck
Fair	Clinical: occasional pain, limited hip movement less than 50%, normal daily activity
	Radiographic: severe deformation of the femoral neck and mild femoral head necrosis
Poor	Clinical: persistent pain, limited hip movement by more than 50%, and limited daily activity
	Radiographic: severe femoral head necrosis, degenerative arthritis, arthrodesis

### Retrospective study

After the approval of the Ethics Committee, a retrospective observational study was conducted based on hospitalized patients and outpatients with AVN after PFNF in our institute from January 2000 to January 2018, according to the following inclusion criteria: (1) participants diagnosed with AVN as a complication of a previous fracture of the femoral neck; (2) patients with no history of corticosteroid administration or alcohol abuse; (3) patients aged less than 17 years when the fracture occurred; (4) patients with no other complications from femoral neck fractures, such as nonunion or infection, or from other diseases, such as dysplasia of the hip joint or rheumatoid arthritis; and (5) patients with complete medical records or radiographic data.

The extracted data consisted of the medical record data and radiographic data. We found the medical records and extracted the following items at the time of initial diagnosis of AVN: (1) demographic data—age, sex, and other personal information; (2) primary clinical data, including symptoms such as pain, limp, and restricted hip function, and the interval between PFNF and AVN; and (3) primary radiographic characteristics of AVN after PFNF, including the stage of disease progression, Japanese Investigation Committee (JIC) classification system [27], and Ratliff classification [2].

The disease progression of AVN was determined according to the Association Research Circulation Osseous staging system [28]: stage I was defined as “normal radiography and computed tomography with an abnormal bone scan and/or magnetic resonance images”; stage II was defined as “sclerosis, osteolysis, or focal osteoporosis in the femoral head”; stage III was defined as “crescent sign and/or flattening of the articular surface” (stage IIIA: collapse < 2 mm, IIIB: collapse ranging from 2 to 4 mm, and IIIC: collapse > 4 mm); and stage IV was defined by the appearance of degenerative changes (osteoarthritis, acetabular changes, or joint destruction). Types A, B, and C1 were assigned to groups where the necrotic area did not extend to the acetabular edge (inside coverage). Type C2 was assigned to groups where there was inside coverage of the necrotic area. Then, we analyzed whether the location of the necrotic area affected the prognosis. The degree of collapse was also measured by evaluating the concentric circles on both anteroposterior and lateral radiographs using ImageJ (1.52a, National Institutes of Health, USA), in reference to a previous study [29].

All the radiographic characteristics and outcomes were evaluated independently by two experienced orthopedic surgeons. If inconsistent results existed, a third surgeon participated and decided the ultimate result.

### Statistical analysis

The relationships between disease progression and other clinical and radiographic factors were analyzed by independent sample *T* tests, Chi-square tests, Fisher’s exact tests, Spearman correlation test, and Mann-Whitney *U* tests. Then, univariate and multivariate analyses were used to detect the OR (odds ratio) and adjusted OR of the factors relevant to the stage of collapse via binary logistic regression models. The variables with *P* < 0.05 were considered significant. The statistical analyses were performed using the SPSS software v.22.0 (SPSS Inc., Chicago, IL, USA).

## Results

### Systematic review

**Fig. 1:**
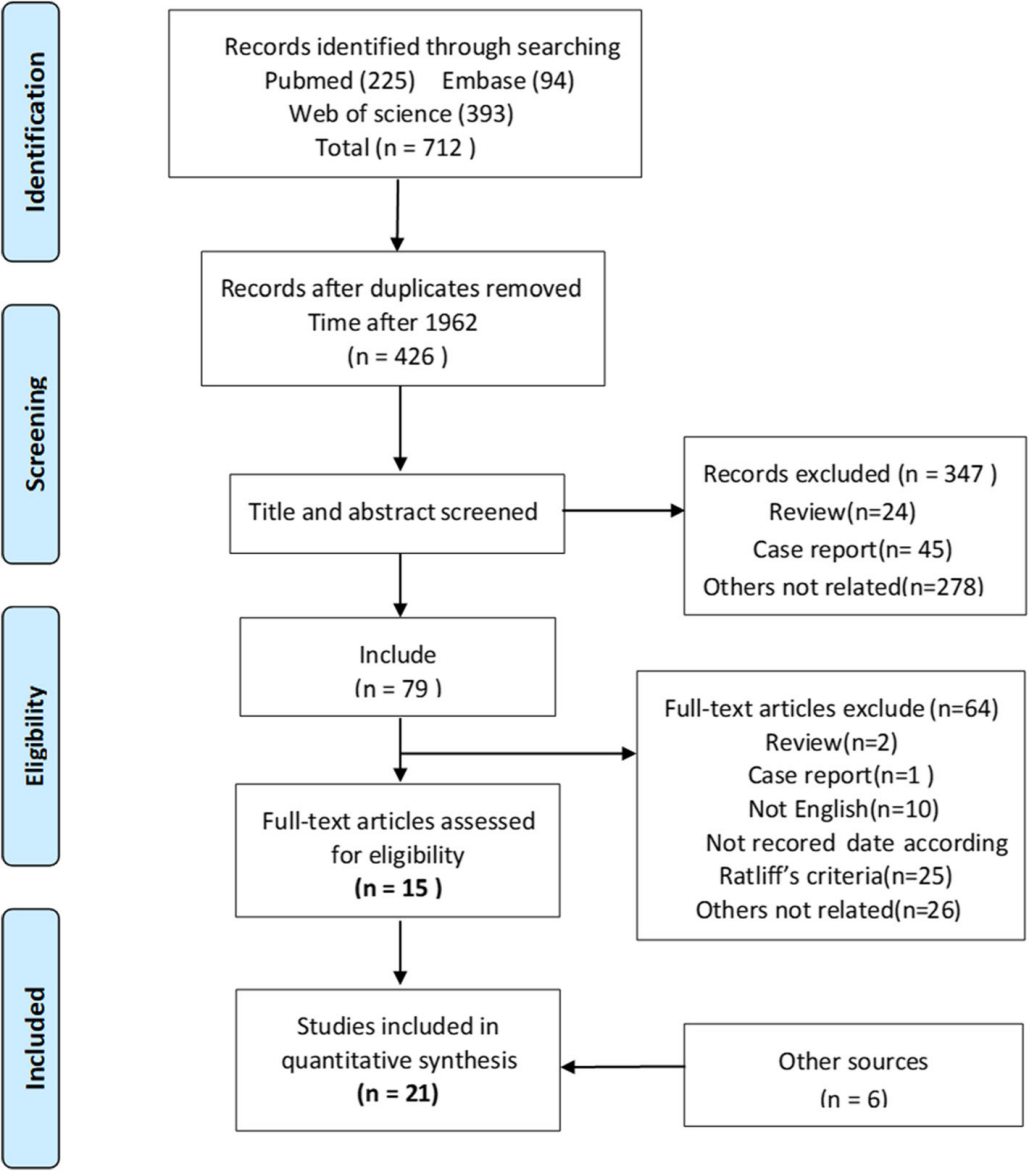
PRISMA flow diagram

Initially, 712 articles were obtained by searching, and two authors obtained 79 studies by reading the title and abstract. Finally, through reading the full text and performing a manual search, a total of 21 articles meeting the requirements were included in our study. The detailed process and information of the 21 included articles are shown in Fig. 1 and Table 1. Finally, 686 patients with PFNF were included. The age range of the patients was 2 to 19 years old. A total of 203 patients developed avascular necrosis, with an incidence of 29.6% (203 of 686 patients). Ratliff’s classification method was used in 19 articles to describe the degree of osteonecrosis, and the classification of osteonecrosis after femoral neck fracture was recorded in 178 pediatric patients, with type I necrosis accounting for 58.4% (104 of 178), type II accounting for 25.3% (45 of 178), and type III accounting for 16.3% (29 of 178). Ratliff’s assessment was used in 13 articles to evaluate the final prognosis of patients from both clinical and imaging perspectives. The final prognosis of osteonecrosis after femoral neck fracture in 147 children was recorded, with 11.6% (17 of 147) having a satisfactory prognosis and 88.4% (130 of 147) having an unsatisfactory prognosis.

### Retrospective study

A total of 155 children and adolescents (155 hips) were diagnosed with AVN after PFNF. In addition, 115 patients had complete medical records or radiographic data. The demographic message of these patients is summarized in Table 3.The mean interval between AVN and PFNF was 13.7 ± 9.5 months. In detail, 71 of 115 (61.7%) cases of AVN were detected in the first year after PFNF, while 32 (27.8%) and 12 (10.4%) were detected within and after the second year, respectively. At the time of diagnosis, 68 were symptomatic patients. The most common symptoms were varying degrees of hip pain, limp, and restricted hip function.

**Table 3:** Demographic, clinical and radiographic characteristics of AVN after PFNF

	Total	Non-collapsed stage	Collapsed stage	***p*** value
**Demographic parameters**				
Age (mean ± SD, years)	13.6 ± 2.0	13.6 ± 2.1	13.5 ± 1.2	0.89*
Sex (*n*, %)				0.72**
Male	78 (67.8)	28 (70.0)	50 (66.7)	
Female	37 (32.2)	12 (30.0)	25 (33.3)	
Side (*n*, %)				< 0.01**
Left	51 (44.3)	11 (27.5)	40 (53.3)	
Right	64 (55.7)	29 (72.5)	35 (46.7)	
**Clinical characteristics**				
Interval between fracture and AVN diagnosis (mean ± SD, months)	13.7 ± 9.5	15.1 ± 9.8	11.2 ± 8.5	0.04*
Symptomatic (*n*, %)				
Yes	68 (59.1)	10 (25.0)	58 (77.3)	< 0.01**
No	47 (40.9)	30 (75.0)	17 (22.7)	
Hip pain (*n*, %)				< 0.01**
Yes	63 (54.8)	10 (25.0)	53 (70.7)	
No	52 (45.2)	30 (75.0)	22 (29.3)	
Limp (*n*, %)				< 0.01**
Yes	58 (50.4)	6 (15.0)	52 (69.3)	
No	57 (49.6)	34 (85.0)	23 (30.7)	
Restricted hip function				< 0.01***
Yes	34 (29.6)	1 (2.5)	33 (56.0)	
No	81 (70.4)	39 (97.5)	42 (44.0)	
**Radiographic characteristics**				
Ratliff classification of AVN (*n*, %)				< 0.01****
Type I	50 (43.5)	7 (17.5)	43 (43.5)	
Type II	61 (53.0)	29 (72.5)	32 (53.0)	
Type III	4 (3.5)	4 (10.0)	0 (0)	
JIC classification of AVN (*n*, %)				< 0.01****
A/B	13 (11.3)	12 (30.0)	1 (1.3)	
C1	40 (34.8)	17 (42.5)	23 (30.7)	
C2	62 (53.9)	11 (27.5)	51 (68.0)	
ARCO stage (*n*, %)				
II	40 (34.8)			
IIIA	34 (29.6)			
IIIB	16 (13.9)			
IIIC	25 (21.7)			

*ARCO* Association Research Circulation Osseous, *AVN* avascular necrosis, *JIC* Japanese Investigation Committee

*Independent sample *t* test

**Chi-square test

***Fisher’s exact test

****Mann-Whitney *U* test

According to the anteroposterior X-ray results and the ACRO staging system, 40 (34.8%) and 75 (65.2%) hips remained with stages II (non-collapsed stage) and III (collapsed stage) disease, respectively; 34 hips collapsed by less than 2 mm (stage IIIA), 16 hips collapsed in a range from 2 to 4 mm (stage IIIB), and 25 hips collapsed by more than 4 mm (stage IIIC). Using Ratliff’s classification, the type III hips (4 hips) were much less than the type I (50 hips) and type II (61 hips). Regarding the JIC classification, the type C2 accounted the most number of included hips (53.9%), followed by the type C1 (34.8%), and type A/B accounted the least part (11.3%).

**Fig. 2:**
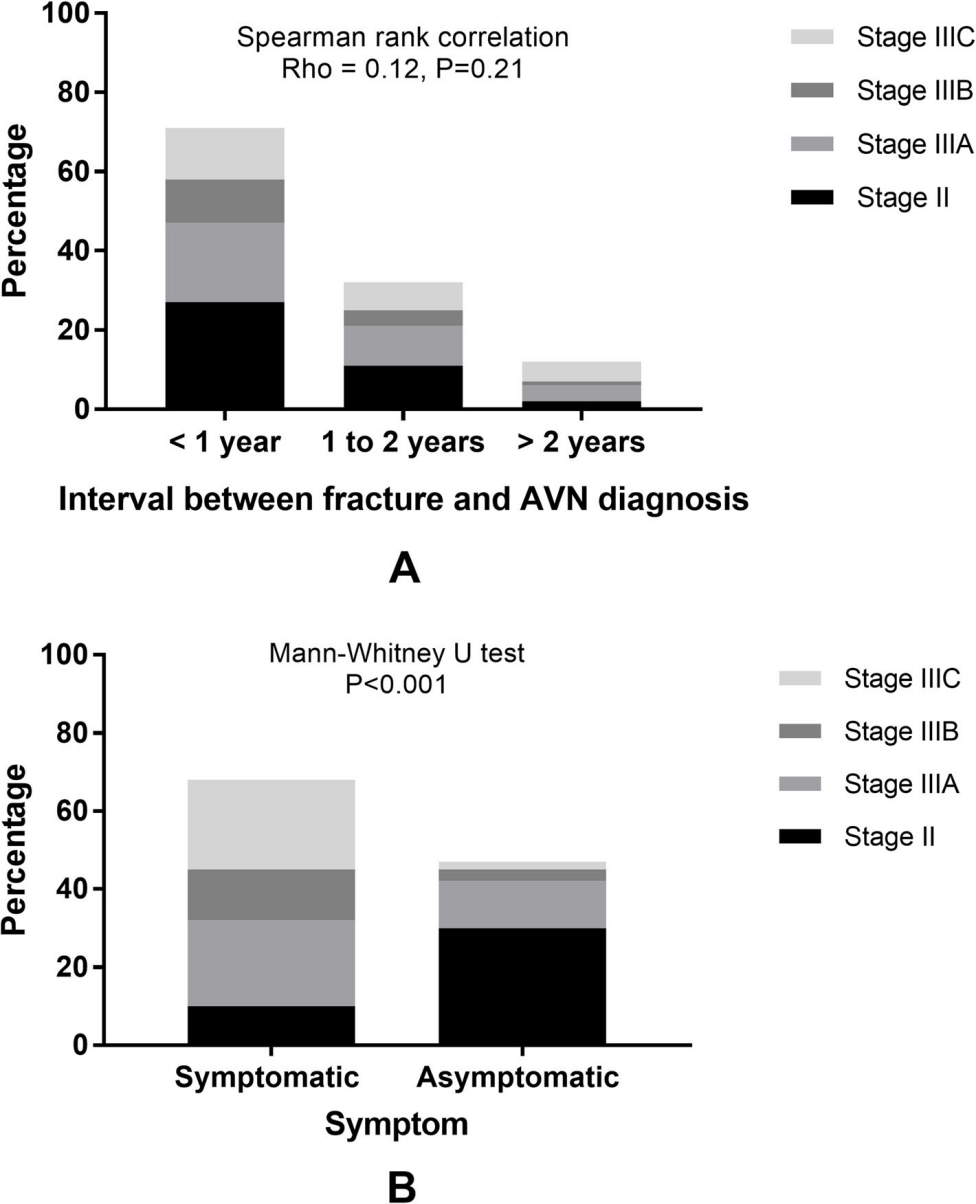
The relationship between clinical symptoms and disease progression

**Fig. 3:**
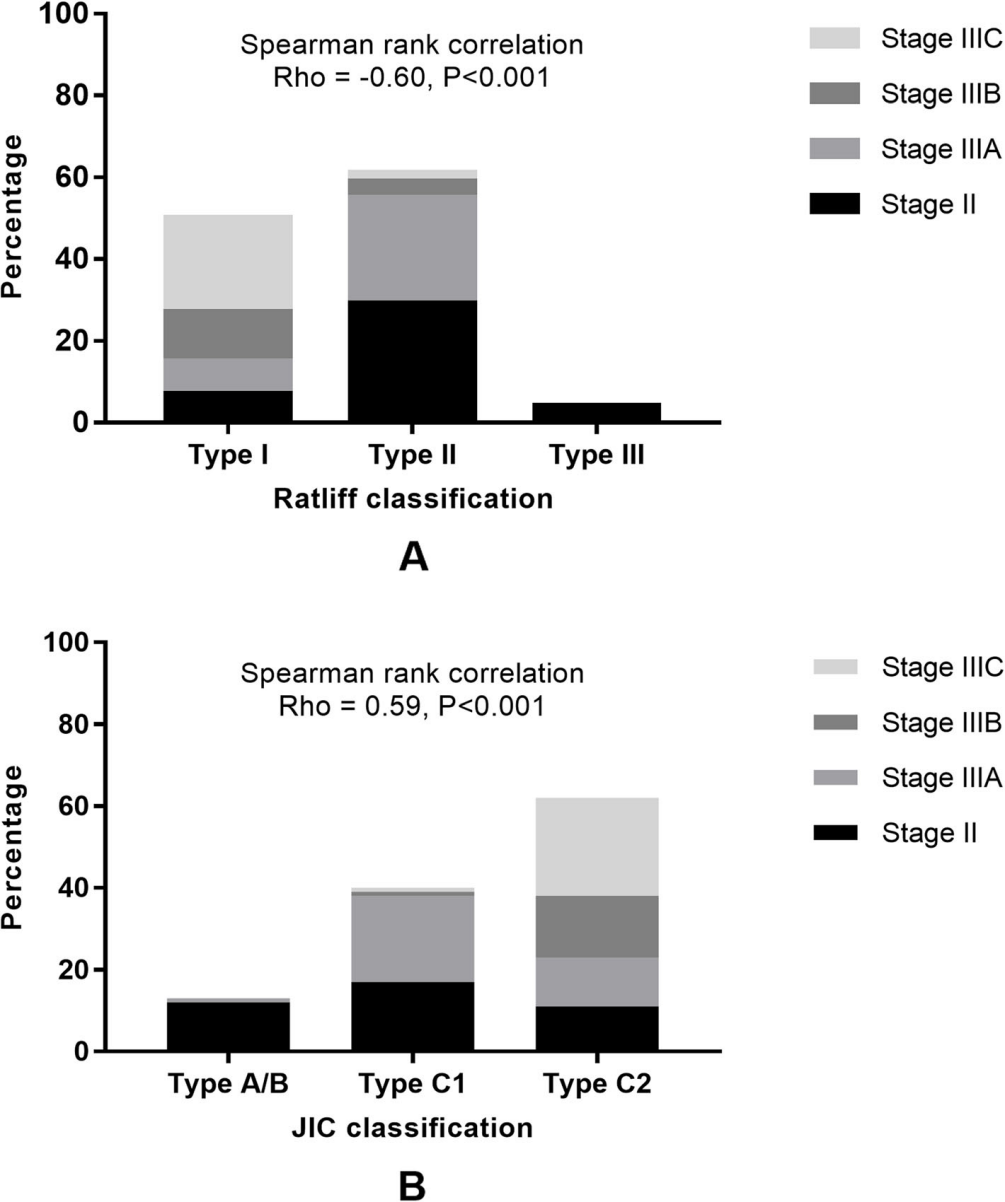
The relationship between JIC classification and Ratliff’s classification and disease progression

The relationships between disease progression, which was defined by ARCO stage, and other clinical and radiographic factors were analyzed (Table 4). Hip symptoms likely indicated a disease progression since the percentage of stage III (collapsed) hips in symptomatic hips (85.3%) is significantly higher than that in asymptomatic hips (36.2%) (Fig. 2). Furthermore, the JIC classification and Ratliff’s classification showed a significant relationship with disease progression (Fig. 3). In detail, the type C2 hips showed the highest risk of collapse progression since 82.3% of them had progressed to femoral head collapse, followed by the type C1 hips (57.5%), and the type A/B showed the lowest risk (7.7%). Not surprisingly, 86% of hips with type I necrosis, which represented the highest risk, were in the collapsed stage, followed by hips with types II (52%) and III (0%) necrosis.

**Table 4:** Relationship between disease characteristics and progression analyzed by Binary logistic regression models

Parameters	ARCO stage					OR (95% CI) of collapsed stage	Adjusted-OR (95% CI) of collapsed stage *
	Total	II	IIIA	IIIB	IIIC		
Age (years, *n*)						0.91 (0.72–1.16)	
≤ 11	17	4	5	3	5		
12	13	3	4	2	4		
13	20	10	4	2	4		
14	25	9	9	2	5		
15	24	8	9	2	5		
≥ 16	16	6	3	5	2		
Sex (*n*)						0.86 (0.37–1.96)	
Male	78	28	25	10	15		
Female	37	12	9	6	10		
Symptomatic (*n*)						10.24 (4.17–25.1) **	6.25 (2.39–16.36) **
No	47	30	12	3	2		
Yes	68	10	22	13	23		
Interval between fracture and AVN diagnosis (*n*)						1.49 (0.81–2.73)	
Within 1 year	71	27	20	11	13		
1 to 2 years	32	11	10	4	7		
More than 2 years	12	2	4	1	5		
JIC classification (*n*)						5.08 (2.54–10.12) **	3.41 (1.62–7.17) **
A/B	13	12	1	0	0		
C1	40	17	21	1	1		
C2	62	11	12	15	24		
Ratliff classification (*n*)						0.15 (0.06–0.37) **	
I	50	7	8	12	23		
II	61	29	26	4	2		
III	4	4	0	0	0		

*ARCO* Association Research Circulation Osseous, *AVN* avascular necrosis, *JIC* Japanese Investigation Committee

*Multivariate analysis with method of “Forward LR”

**p < 0.05

Unadjusted univariate analysis was used to detect the odds ratio (OR). Disease stage presented no significant correlation with age (OR = 0.91, 95% CI = 0.72–1.16), sex (OR = 0.86, 95% CI = 0.37–1.96), and interval between fracture and AVN diagnosis (OR = 1.49, 95% CI = 0.81–2.73), however, a significant relation with symptom (OR = 10.24, 95% CI = 4.17–25.1), JIC classification (OR = 5.08, 95% CI = 2.54–10.12), and Ratliff classification (OR = 0.15, 95% CI = 0.06–0.37). Then, multivariate analysis was used to detect the adjusted OR of the factors relevant to the stage of collapse. Symptomatic patients (OR = 6.25, 95% CI = 2.39–16.36) and JIC classification (OR = 3.41, 95% CI = 1.62–7.17) showed a strong relationship with the stage of collapse in AVN.

## Discussion

A classification system and a set of criteria for clinical and radiographic assessment were first reported by Ratliff and his colleagues in the 1960s, portraying AVN as a severe complication secondary to pediatric femoral neck fractures [2]. Since then, numerous studies have adapted Ratliff’s methods described above to classify AVN after PFNF and to assess outcomes. However, owing to the limitation of sample size, the specific characteristics of AVN after PFNF remain unknown. This study is the first, to our knowledge, to address these deficiencies through a cross-sectional study of diagnostic data from 115 patients and a systematic review.

The best characteristic for predicting AVN after PFNF was a rapid disease course with a high risk of femoral head collapse and poorer prognosis. As the first step, we identified similar data for a systematic review. Among the 21 studies enrolled, 19 adopted Ratliff’s classification and included 178 hips, with types I, II, and III necrosis 58.4%, 25.3%, and 16.3%, respectively, and the final prognosis of AVN after PFNF in 147 children was recorded, 88.4% having an unsatisfactory prognosis. Among the 115 hips from our clinical data, 43.5%, 53.0%, and 3.5% were defined as having types I, II, and III necrosis, respectively, according to Ratliff’s classification. A systematic review and our clinical data indicated a dominant proportion of extensively involved AVN lesions after PFNF, inevitably pointing to the rapid progression of the disease. As Ratliff reported [2], patients with type I and type II AVN are generally predisposed to a poor prognosis, often with progressive femoral head collapse and hip subluxation and, ultimately, hip degeneration. No hip-preserving treatments with confirmed therapeutic effects except for arthrodesis or arthroplasty have been recommended for these severe conditions [21, 30]. According to the mainstream explanations of the published literature, this poor condition can primarily be ascribed to the high-energy primary trauma and the obstruction of compensatory blood supplies induced by immature epiphyseal plates in these populations [1, 2, 31]. Similarly, Legg-Calve-Perthes disease is also a common etiology of childhood osteonecrosis, usually with extensive and severe involvement of the epiphysis [32, 33].

Symptoms, as one of the recognized prognostic factors of disease progression in most types of AVN, showed a significant relationship with the ARCO stage in this study [26, 34, 35]. According to our data, hip symptoms, such as hip pain, limp, and restricted hip function, were recorded in 68 of 115 cases, 85.3% of which had progressed to femoral head collapse. In contrast, 47 asymptomatic patients were diagnosed via routine follow-up, and only 36.2% of them had already progressed to the collapsed stage. These data likely suggest symptoms as a risk factor for femoral head collapse. On the other hand, the interval between hip fracture and AVN diagnosis was recorded. The average duration was 13.7 months, which was similar to a previous report [36]. Considering that 61.7% and 27.8% of AVN cases were diagnosed in the first and second years after a hip injury, a prolonged follow-up of 2 years was indispensable for this population, even for asymptomatic cases.

**Fig. 4:**
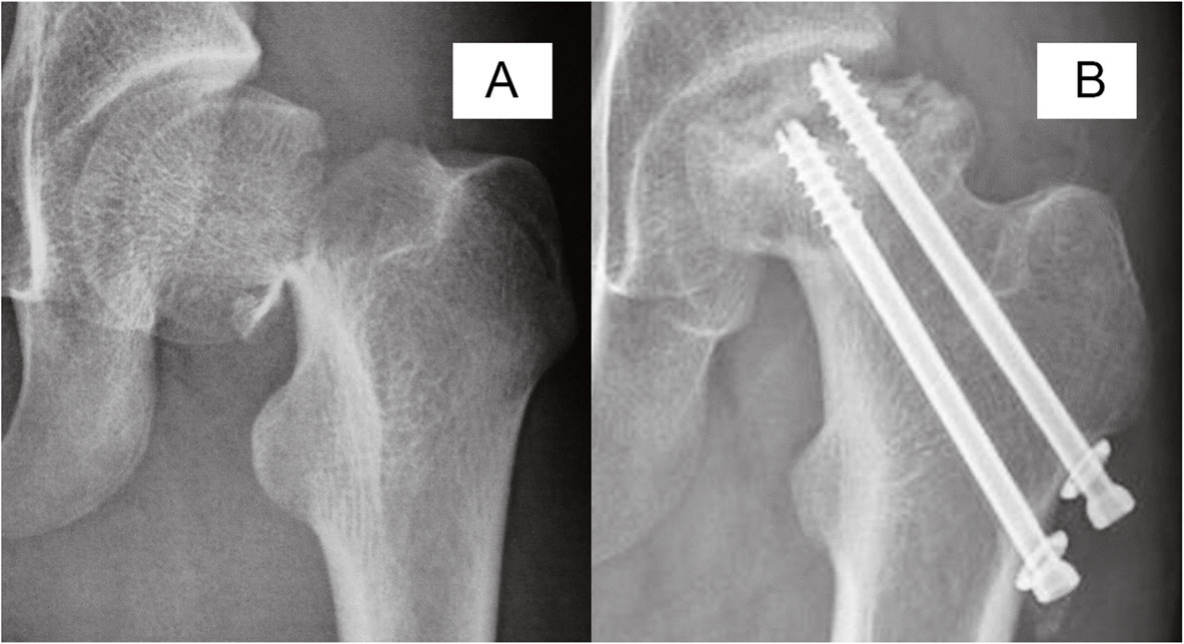
The anteroposterior radiographs of Ratliff type I avascular necrosis after pediatric femoral neck fracture. Femoral neck fracture occurred at age of 14 years (**a**) and avascular necrosis was diagnosed 16 months later (**b**), type C2 according JIC classification, presenting severe femoral head and hip subluxation

**Fig. 5:**
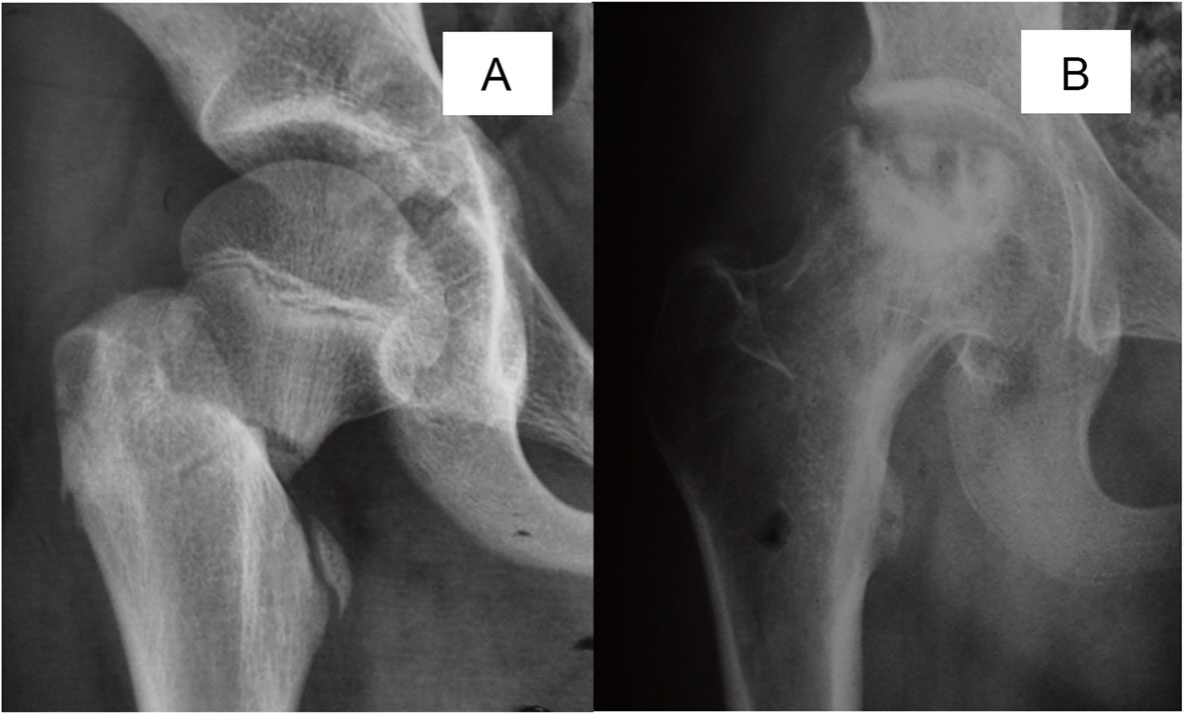
The anteroposterior radiographs of Ratliff type I avascular necrosis after pediatric femoral neck fracture. Femoral neck fracture occurred at age of 12 years (**a**) and avascular necrosis were diagnosed 8 months later (**b**), and JIC type C2, presenting collapsed femoral head

**Fig. 6:**
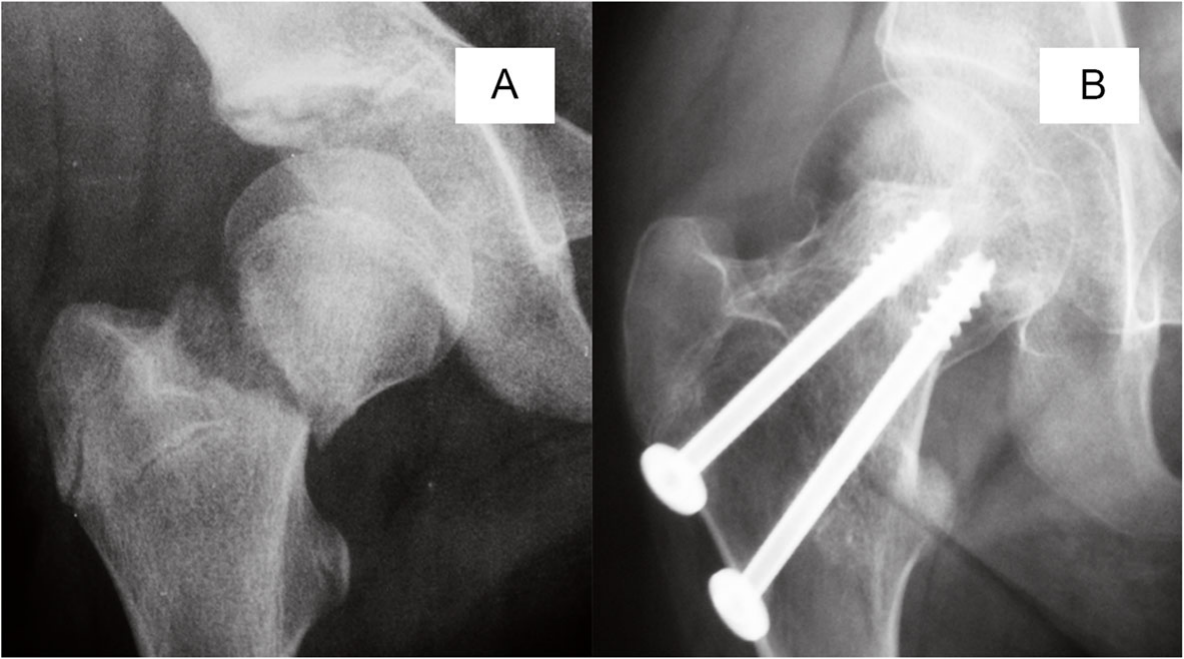
The anteroposterior radiographs of Ratliff type II avascular necrosis after pediatric femoral neck fracture. Femoral neck fracture occurred at age of 10 years (**a**) and avascular necrosis were diagnosed 5 months later (**b**), and JIC type C1, presenting non-collapsed femoral head

As a cross-sectional study, our data revealed a potential relationship between disease progression and necrotic involvement. We found that at the time of diagnosis, 43 of 50 (86%) hips with type I necrosis and 32 of 61 (52%) with type II necrosis had already progressed to femoral head collapse, and there was no collapsed hip in type III, indicating that in the type I hips appear the higher risk of collapse compared with other two types. The influence of the location of the lesion is not demonstrated in the Ratliff classification. Therefore, we attempted to use the JIC classification as a complement to address the deficiency of Ratliff’s classification, principally as a result of setting a subclassification of Ratliff’s type II AVN. It is widely accepted that the JIC classification is a practical method for predicting the risk of femoral head collapse in adult necrosis of the femoral head with confirmed intra- and interobserver concordance [37]. There is no doubt that all the cases of Ratliff’s type I AVN were classified as JIC stage C2 (Fig. 4); however, the definition of Ratliff’s type II AVN is vague. The partial involvement of necrosis can also be classified as JIC stage C1 or C2 (Figs. 5 and 6). Both of these conditions involve the lateral part of the femoral head; however, in the latter stage, AVN encroaches extensively beyond the lateral margin of the acetabulum and induces the highest risk of femoral head collapse. In the current study, at the time of AVN diagnosis, correlation analysis indicated a significant positive relationship between disease stage and JIC classification. In detail, 82.3% of type C2 hips and 57.5% of type C1 hips progressed to femoral head collapse. Further multivariate logistic analysis also demonstrated that the JIC classification showed a stronger correlation with femoral head collapse than did Ratliff’s classification.

Several limitations still exist. First and foremost, although this investigation was the first, to our knowledge, to include the largest sample size of enrolled patients to describe the radiographic and clinical characteristics of AVN after PFNF via a cross-sectional study, patient selection bias should not be neglected in a retrospective study. Secondly, as a retrospective study, we failed to record or analyze the factors related to primary hip fracture in all patients, such as the classification, degree of displacement, methods of reduction, and types of internal fixation. Lastly, as a cross-sectional study, although our data related radiographic and clinical characteristics to disease progression, we could not confirm the prognostic value of these factors. Further prospective multicenter control trials or case series with advanced radiologic technology are suggested to confirm the results.

## Conclusions

In summary, our recent study first identified the clinical and radiographic characteristics of AVN after PFNF. According to our results from a systematic review and cross-sectional study, we believe that the most prominent feature of AVN after PFNF is the tendency toward extensive necrotic lesions, which predisposes this population to a poor prognosis. More than half of the patients had progressed to an advanced stage when the diagnosis of AVN was confirmed, usually around the first year after PFNF. And the symptoms and the JIC classification are the other two risk factors of collapse progression.

## Supplementary Information

**Additional file 1.** Search strategy.

**Additional file 2.** IHE’s quality appraisal checklist for assessing case-series studies.

## Data Availability

The authors declare that all the data supporting the findings of this study are available within the article and its supplementary information files.

## Abbreviations

PFNFs: Pediatric femoral neck fractures
AVN: Avascular necrosis
IHE: Institute of Health Economics
JIC: Japanese Investigation Committee
ARCO: Association Research Circulation Osseous
OR: Odds ratio
