# Supplementary material for: The clinical and radiographic characteristics of avascular necrosis after pediatric femoral neck fracture: a systematic review and retrospective study of 115 patients
Source: J Orthop Surg Res. 2020 Nov 11;15:520. doi: 10.1186/s13018-020-02037-2 (PMC7661253; doi:10.1186/s13018-020-02037-2)
Supplement: Supplementary file 1 — Additional file 1. Search strategy. [file 13018_2020_2037_MOESM1_ESM.docx]

| Embase |  |  |  |  |  |  |
| --- | --- | --- | --- | --- | --- | --- |
| Session Results |  |  |  |  |  |  |
| ....................................................... |  |  |  |  |  |  |
| No. Query Results Results Date |  |  |  |  |  |  |
| #40. #22 AND #27 AND #39 94 24 Sep 2019 |  |  |  |  |  |  |
| #39. #28 OR #29 OR #30 OR #31 OR #32 OR #33 OR #34 OR 5 | 901 24 Sep 2019 |  |  |  |  |  |
| #35 OR #36 OR #37 OR #38 |  |  |  |  |  |  |
| #38. 'avascular necrosis of femur head':ab | ti 11 24 Sep 2019 |  |  |  |  |  |
| #37. 'avascular necrosis of femoral head | 24 Sep 2019 |  |  |  |  |  |
| primary':ab | ti |  |  |  |  |  |
| #36. 'femoral head | avascular necrosis of':ab | ti 3 24 Sep 2019 |  |  |  |  |
| #35. 'ischemic necrosis of femoral head':ab | ti 38 24 Sep 2019 |  |  |  |  |  |
| #34. 'necrosis | avascular | of femur head':ab | ti 24 Sep 2019 | | |  |
| #33. 'necrosis | aseptic | of femur head':ab | ti 24 Sep 2019 | | |  |
| #32. 'aseptic necrosis of femur head':ab | ti 6 24 Sep 2019 |  |  |  |  |  |
| #31. 'necrosis | femur head':ab | ti 24 Sep 2019 |  |  |  |  |
| #30. 'head necrosis | femur':ab | ti 24 Sep 2019 |  |  |  |  |
| #29. 'femur head necroses':ab | ti 11 24 Sep 2019 |  |  |  |  |  |
| #28. 'femur head necrosis'/exp 5 | 872 24 Sep 2019 |  |  |  |  |  |
| #27. #23 OR #24 OR #25 OR #26 12 | 219 24 Sep 2019 |  |  |  |  |  |
| #26. 'femur neck fracture':ab | ti 103 24 Sep 2019 |  |  |  |  |  |
| #25. 'femur neck fractures':ab | ti 114 24 Sep 2019 |  |  |  |  |  |
| #24. 'femoral neck fracture':ab | ti 3 | 195 24 Sep 2019 |  |  |  |  |
| #23. 'femoral neck fracture'/exp 11 | 754 24 Sep 2019 |  |  |  |  |  |
| #22. #3 OR #4 OR #5 OR #7 OR #8 OR #9 OR #10 OR #11 OR 3 | 876 | 311 24 Sep 2019 |  |  |  |  |
| #12 OR #13 OR #14 OR #15 OR #16 OR #17 OR #18 OR |  |  |  |  |  |  |
| #19 OR #20 OR #21 |  |  |  |  |  |  |
| #21. 'male adolescents':ab | ti 2 | 702 24 Sep 2019 |  |  |  |  |
| #20. 'male adolescent':ab | ti 1 | 055 24 Sep 2019 |  |  |  |  |
| #19. 'adolescent | male':ab | ti 1 | 970 24 Sep 2019 | |  |  |
| #18. 'adolescents | male':ab | ti 103 24 Sep 2019 |  |  |  |  |
| #17. 'female adolescents':ab | ti 4 | 537 24 Sep 2019 |  |  |  |  |
| #16. 'female adolescent':ab | ti 1 | 389 24 Sep 2019 |  |  |  |  |
| #15. 'adolescent | female':ab | ti 1 | 814 24 Sep 2019 | |  |  |
| #14. 'adolescents | female':ab | ti 48 24 Sep 2019 |  |  |  |  |
| #13. 'youths':ab | ti 12 | 138 24 Sep 2019 |  |  |  |  |
| #12. 'youth':ab | ti 74 | 768 24 Sep 2019 |  |  |  |  |
| #11. 'teenager':ab | ti 3 | 882 24 Sep 2019 |  |  |  |  |
| #10. 'teenagers':ab | ti 16 | 076 24 Sep 2019 |  |  |  |  |
| #9. 'teen':ab | ti 6 | 681 24 Sep 2019 |  |  |  |  |
| #8. 'teens':ab | ti 8 | 341 24 Sep 2019 |  |  |  |  |
| #7. 'adolescence':ab | ti 67 | 397 24 Sep 2019 |  |  |  |  |
| #6. 'adolescence teens':ab | ti 6 24 Sep 2019 |  |  |  |  |  |
| #5. 'adolescents':ab | ti 227 | 322 24 Sep 2019 |  |  |  |  |
| #4. 'adolescent'/exp 1 | 602 | 662 24 Sep 2019 |  |  |  |  |
| #3. #1 OR #2 3 | 66 | 653 24 Sep 2019 |  |  |  |  |
| #2. 'children':ab | ti 1 | 333 | 051 24 Sep 2019 | |  |  |
| #1. 'child'/exp 2 | 777 | 139 24 Sep 2019 |  |  |  |  |
| ....................................................... |  |  |  |  |  |  |
|  |  |  |  |  |  |  |

Pubmed history

| Recent queries in pubmed | |  |  |
| --- | --- | --- | --- |
| Search | Query | Items found | Time |
| #26 | Search ((((((((((((((((((((((Adolescents[Title/Abstract]) OR Adolescence[Title/Abstract]) OR Teens[Title/Abstract]) OR Teen[Title/Abstract]) OR Teenagers[Title/Abstract]) OR Teenager[Title/Abstract]) OR Youth[Title/Abstract]) OR Youths[Title/Abstract]) OR Adolescents, Female[Title/Abstract]) OR Adolescent, Female[Title/Abstract]) OR Female Adolescent[Title/Abstract]) OR Female Adolescents[Title/Abstract]) OR Adolescents, Male[Title/Abstract]) OR Adolescent, Male[Title/Abstract]) OR Male Adolescent[Title/Abstract]) OR Male Adolescents[Title/Abstract])) OR "Adolescent"[Mesh])) OR ((children[Title/Abstract]) OR "Child"[Mesh]))) AND (((((Femoral Neck Fracture[Title/Abstract]) OR Femur Neck Fractures[Title/Abstract]) OR Femur Neck Fracture[Title/Abstract])) OR "Femoral Neck Fractures"[Mesh])) AND ((((((((((((Femur Head Necroses[Title/Abstract]) OR Head Necrosis, Femur[Title/Abstract]) OR Necrosis, Femur Head[Title/Abstract]) OR Aseptic Necrosis of Femur Head[Title/Abstract]) OR Necrosis, Aseptic, of Femur Head[Title/Abstract]) OR Necrosis, Avascular, of Femur Head[Title/Abstract]) OR Ischemic Necrosis Of Femoral Head[Title/Abstract]) OR Femoral Head, Avascular Necrosis Of[Title/Abstract]) OR Avascular Necrosis Of Femoral Head, Primary[Title/Abstract]) OR Avascular Necrosis of Femur Head[Title/Abstract])) OR "Femur Head Necrosis"[Mesh]) | 225 | 22:07:50 |
| #25 | Search (((((((((((Femur Head Necroses[Title/Abstract]) OR Head Necrosis, Femur[Title/Abstract]) OR Necrosis, Femur Head[Title/Abstract]) OR Aseptic Necrosis of Femur Head[Title/Abstract]) OR Necrosis, Aseptic, of Femur Head[Title/Abstract]) OR Necrosis, Avascular, of Femur Head[Title/Abstract]) OR Ischemic Necrosis Of Femoral Head[Title/Abstract]) OR Femoral Head, Avascular Necrosis Of[Title/Abstract]) OR Avascular Necrosis Of Femoral Head, Primary[Title/Abstract]) OR Avascular Necrosis of Femur Head[Title/Abstract])) OR "Femur Head Necrosis"[Mesh] | 8462 | 22:04:51 |
| #24 | Search (((((((((Femur Head Necroses[Title/Abstract]) OR Head Necrosis, Femur[Title/Abstract]) OR Necrosis, Femur Head[Title/Abstract]) OR Aseptic Necrosis of Femur Head[Title/Abstract]) OR Necrosis, Aseptic, of Femur Head[Title/Abstract]) OR Necrosis, Avascular, of Femur Head[Title/Abstract]) OR Ischemic Necrosis Of Femoral Head[Title/Abstract]) OR Femoral Head, Avascular Necrosis Of[Title/Abstract]) OR Avascular Necrosis Of Femoral Head, Primary[Title/Abstract]) OR Avascular Necrosis of Femur Head[Title/Abstract] | 2299 | 22:03:41 |
| #23 | Search "Femur Head Necrosis"[Mesh] | 7687 | 21:59:45 |
| #21 | Search ((((Femoral Neck Fracture[Title/Abstract]) OR Femur Neck Fractures[Title/Abstract]) OR Femur Neck Fracture[Title/Abstract])) OR "Femoral Neck Fractures"[Mesh] | 9577 | 21:58:46 |
| #20 | Search ((Femoral Neck Fracture[Title/Abstract]) OR Femur Neck Fractures[Title/Abstract]) OR Femur Neck Fracture[Title/Abstract] | 3342 | 21:58:35 |
| #18 | Search "Femoral Neck Fractures"[Mesh] | 8553 | 21:57:54 |
| #16 | Search (((((((((((((((((((Adolescents[Title/Abstract]) OR Adolescence[Title/Abstract]) OR Teens[Title/Abstract]) OR Teen[Title/Abstract]) OR Teenagers[Title/Abstract]) OR Teenager[Title/Abstract]) OR Youth[Title/Abstract]) OR Youths[Title/Abstract]) OR Adolescents, Female[Title/Abstract]) OR Adolescent, Female[Title/Abstract]) OR Female Adolescent[Title/Abstract]) OR Female Adolescents[Title/Abstract]) OR Adolescents, Male[Title/Abstract]) OR Adolescent, Male[Title/Abstract]) OR Male Adolescent[Title/Abstract]) OR Male Adolescents[Title/Abstract])) OR "Adolescent"[Mesh])) OR ((children[Title/Abstract]) OR "Child"[Mesh]) | 3213689 | 21:55:10 |
| #15 | Search (((((((((((((((((Adolescents[Title/Abstract]) OR Adolescence[Title/Abstract]) OR Teens[Title/Abstract]) OR Teen[Title/Abstract]) OR Teenagers[Title/Abstract]) OR Teenager[Title/Abstract]) OR Youth[Title/Abstract]) OR Youths[Title/Abstract]) OR Adolescents, Female[Title/Abstract]) OR Adolescent, Female[Title/Abstract]) OR Female Adolescent[Title/Abstract]) OR Female Adolescents[Title/Abstract]) OR Adolescents, Male[Title/Abstract]) OR Adolescent, Male[Title/Abstract]) OR Male Adolescent[Title/Abstract]) OR Male Adolescents[Title/Abstract])) OR "Adolescent"[Mesh] | 2036631 | 21:54:35 |
| #14 | Search (((((((((((((((Adolescents[Title/Abstract]) OR Adolescence[Title/Abstract]) OR Teens[Title/Abstract]) OR Teen[Title/Abstract]) OR Teenagers[Title/Abstract]) OR Teenager[Title/Abstract]) OR Youth[Title/Abstract]) OR Youths[Title/Abstract]) OR Adolescents, Female[Title/Abstract]) OR Adolescent, Female[Title/Abstract]) OR Female Adolescent[Title/Abstract]) OR Female Adolescents[Title/Abstract]) OR Adolescents, Male[Title/Abstract]) OR Adolescent, Male[Title/Abstract]) OR Male Adolescent[Title/Abstract]) OR Male Adolescents[Title/Abstract] | 418688 | 21:54:10 |
| #12 | Search "Adolescent"[Mesh] | 1957740 | 21:48:27 |
| #9 | Search (children[Title/Abstract]) OR "Child"[Mesh] | 2125764 | 21:47:12 |
| #8 | Search children[Title/Abstract] | 988235 | 21:41:31 |
| #3 | Search "Child"[Mesh] | 1850045 | 21:12:53 |

Web of science history

| <search> |  |  |  |  |  |  |  |  |  |
| --- | --- | --- | --- | --- | --- | --- | --- | --- | --- |
| <application_name>UA</application_name> | | | | |  |  |  |  |  |
| <application_version>WoK 5.5</application_version> | | | | |  |  |  |  |  |
| <search_name>Test Search</search_name> | | | | |  |  |  |  |  |
| <search_desc>Test Search Description</search_desc> | | | | | |  |  |  |  |
| <sets> |  |  |  |  |  |  |  |  |  |
| <set> |  |  |  |  |  |  |  |  |  |
| <set_number>1</set_number> | | |  |  |  |  |  |  |  |
| <field_id></field_id> | |  |  |  |  |  |  |  |  |
| <tns_id></tns_id> | |  |  |  |  |  |  |  |  |
| <parsed_query_string><![CDATA[]]></parsed_query_string> | | | | | |  |  |  |  |
| <user_query_string><![CDATA[TS=child*]]></user_query_string> | | | | | | |  |  |  |
| <user_timespan_string><![CDATA[1962-2019]]></user_timespan_string> | | | | | | |  |  |  |
| <parsed_dblimits_string><![CDATA[WOS.IC WOS.SCI WOS.CCR KJD.KJD MEDLINE.MEDLINE RSCI.RSCI SCIELO.SCIELO]]></parsed_dblimits_string> | | | | | | | | | |
| <user_editions_string><![CDATA[ WOS | KJD | MEDLINE | RSCI | SCIELO]]></user_editions_string> | | | |  |  |
| <user_limits_string><![CDATA[]]></user_limits_string> | | | | | |  |  |  |  |
| <refinedBy><![CDATA[]]></refinedBy> | | | |  |  |  |  |  |  |
| <analysisHistory></analysisHistory> | | | |  |  |  |  |  |  |
| <application_data> | |  |  |  |  |  |  |  |  |
| <service_mode></service_mode> | | | |  |  |  |  |  |  |
| <query_desc>AdvancedSearch</query_desc> | | | | |  |  |  |  |  |
| <query_type>com.thomson.ts.framework.query.IGeneralQuery</query_type> | | | | | | | |  |  |
| <preparsed><![CDATA[TS=child* ]]></preparsed> | | | | |  |  |  |  |  |
| <molecule><![CDATA[]]></molecule> | | | |  |  |  |  |  |  |
| <frag_codes><![CDATA[]]></frag_codes> | | | |  |  |  |  |  |  |
| <queryType>com.thomson.ts.framework.query.IGeneralQuery</queryType> | | | | | | | |  |  |
| <query_syntax>3.3</query_syntax> | | | |  |  |  |  |  |  |
| <oldQuerySyntax></oldQuerySyntax> | | | |  |  |  |  |  |  |
| <search_mode>AdvancedSearch</search_mode> | | | | |  |  |  |  |  |
| <colName></colName> | | |  |  |  |  |  |  |  |
| <unparsedSearchInput><![CDATA[TS=child* ]]></unparsedSearchInput> | | | | | | |  |  |  |
| <parsedSearchInput><![CDATA[]]></parsedSearchInput> | | | | | |  |  |  |  |
| <unparsedSearchNoLimits><![CDATA[TS=child* ]]></unparsedSearchNoLimits> | | | | | | | |  |  |
| <subtype></subtype> | | |  |  |  |  |  |  |  |
| <options></options> | | |  |  |  |  |  |  |  |
| <user_query_string_xml><Field><![CDATA[TS=child*]]></Field></user_query_string_xml> | | | | | | | | |  |
| <ss_lemmatization><![CDATA[On]]></ss_lemmatization> | | | | | |  |  |  |  |
| <ss_spellchecking><![CDATA[Suggest]]></ss_spellchecking> | | | | | |  |  |  |  |
| <ss_query_language><![CDATA[auto]]></ss_query_language> | | | | | |  |  |  |  |
| <isOneClickDaisyAuthor></isOneClickDaisyAuthor> | | | | |  |  |  |  |  |
| <OneClickDaisyAuthorName></OneClickDaisyAuthorName> | | | | | |  |  |  |  |
| <relatedMetaData></relatedMetaData> | | | |  |  |  |  |  |  |
| <parentUT></parentUT> | | |  |  |  |  |  |  |  |
| </application_data> | |  |  |  |  |  |  |  |  |
| </set> |  |  |  |  |  |  |  |  |  |
| <set> |  |  |  |  |  |  |  |  |  |
| <set_number>2</set_number> | | |  |  |  |  |  |  |  |
| <field_id></field_id> | |  |  |  |  |  |  |  |  |
| <tns_id></tns_id> | |  |  |  |  |  |  |  |  |
| <parsed_query_string><![CDATA[]]></parsed_query_string> | | | | | |  |  |  |  |
| <user_query_string><![CDATA[TS=（adolescen* or Teen* or Teenager* or Youth* or Adolescents | Female or Adolescent | Female or Female Adolescents or Adolescents | Male or Adolescent | Male or Male Adolescent or Male Adolescents）]]></user_query_string> | | | | | |
| <user_timespan_string><![CDATA[1962-2019]]></user_timespan_string> | | | | | | |  |  |  |
| <parsed_dblimits_string><![CDATA[WOS.IC WOS.SCI WOS.CCR KJD.KJD MEDLINE.MEDLINE RSCI.RSCI SCIELO.SCIELO]]></parsed_dblimits_string> | | | | | | | | | |
| <user_editions_string><![CDATA[ WOS | KJD | MEDLINE | RSCI | SCIELO]]></user_editions_string> | | | |  |  |
| <user_limits_string><![CDATA[]]></user_limits_string> | | | | | |  |  |  |  |
| <refinedBy><![CDATA[]]></refinedBy> | | | |  |  |  |  |  |  |
| <analysisHistory></analysisHistory> | | | |  |  |  |  |  |  |
| <application_data> | |  |  |  |  |  |  |  |  |
| <service_mode></service_mode> | | | |  |  |  |  |  |  |
| <query_desc>AdvancedSearch</query_desc> | | | | |  |  |  |  |  |
| <query_type>com.thomson.ts.framework.query.IGeneralQuery</query_type> | | | | | | | |  |  |
| <preparsed><![CDATA[TS=（adolescen* or Teen* or Teenager* or Youth* or Adolescents | Female or Adolescent | Female or Female Adolescents or Adolescents | Male or Adolescent | Male or Male Adolescent or Male Adolescents） ]]></preparsed> | | | | | |
| <molecule><![CDATA[]]></molecule> | | | |  |  |  |  |  |  |
| <frag_codes><![CDATA[]]></frag_codes> | | | |  |  |  |  |  |  |
| <queryType>com.thomson.ts.framework.query.IGeneralQuery</queryType> | | | | | | | |  |  |
| <query_syntax>3.3</query_syntax> | | | |  |  |  |  |  |  |
| <oldQuerySyntax></oldQuerySyntax> | | | |  |  |  |  |  |  |
| <search_mode>AdvancedSearch</search_mode> | | | | |  |  |  |  |  |
| <colName></colName> | | |  |  |  |  |  |  |  |
| <unparsedSearchInput><![CDATA[TS=（adolescen* or Teen* or Teenager* or Youth* or Adolescents | Female or Adolescent | Female or Female Adolescents or Adolescents | Male or Adolescent | Male or Male Adolescent or Male Adolescents） ]]></unparsedSearchInput> | | | | | |
| <parsedSearchInput><![CDATA[]]></parsedSearchInput> | | | | | |  |  |  |  |
| <unparsedSearchNoLimits><![CDATA[TS=（adolescen* or Teen* or Teenager* or Youth* or Adolescents | Female or Adolescent | Female or Female Adolescents or Adolescents | Male or Adolescent | Male or Male Adolescent or Male Adolescents） ]]></unparsedSearchNoLimits> | | | | | |
| <subtype></subtype> | | |  |  |  |  |  |  |  |
| <options></options> | | |  |  |  |  |  |  |  |
| <user_query_string_xml><Field><![CDATA[TS=（adolescen* or Teen* or Teenager* or Youth* or Adolescents | Female or Adolescent | Female or Female Adolescents or Adolescents | Male or Adolescent | Male or Male Adolescent or Male Adolescents）]]></Field></user_query_string_xml> | | | | | |
| <ss_lemmatization><![CDATA[On]]></ss_lemmatization> | | | | | |  |  |  |  |
| <ss_spellchecking><![CDATA[Suggest]]></ss_spellchecking> | | | | | |  |  |  |  |
| <ss_query_language><![CDATA[auto]]></ss_query_language> | | | | | |  |  |  |  |
| <isOneClickDaisyAuthor></isOneClickDaisyAuthor> | | | | |  |  |  |  |  |
| <OneClickDaisyAuthorName></OneClickDaisyAuthorName> | | | | | |  |  |  |  |
| <relatedMetaData></relatedMetaData> | | | |  |  |  |  |  |  |
| <parentUT></parentUT> | | |  |  |  |  |  |  |  |
| </application_data> | |  |  |  |  |  |  |  |  |
| </set> |  |  |  |  |  |  |  |  |  |
| <set> |  |  |  |  |  |  |  |  |  |
| <set_number>3</set_number> | | |  |  |  |  |  |  |  |
| <field_id></field_id> | |  |  |  |  |  |  |  |  |
| <tns_id></tns_id> | |  |  |  |  |  |  |  |  |
| <parsed_query_string><![CDATA[]]></parsed_query_string> | | | | | |  |  |  |  |
| <user_query_string><![CDATA[TS=（femoral neck fracture or Femoral Neck Fracture or Femur Neck Fractures or Femur Neck Fracture ）]]></user_query_string> | | | | | | | | | |
| <user_timespan_string><![CDATA[1962-2019]]></user_timespan_string> | | | | | | |  |  |  |
| <parsed_dblimits_string><![CDATA[WOS.IC WOS.SCI WOS.CCR KJD.KJD MEDLINE.MEDLINE RSCI.RSCI SCIELO.SCIELO]]></parsed_dblimits_string> | | | | | | | | | |
| <user_editions_string><![CDATA[ WOS | KJD | MEDLINE | RSCI | SCIELO]]></user_editions_string> | | | |  |  |
| <user_limits_string><![CDATA[]]></user_limits_string> | | | | | |  |  |  |  |
| <refinedBy><![CDATA[]]></refinedBy> | | | |  |  |  |  |  |  |
| <analysisHistory></analysisHistory> | | | |  |  |  |  |  |  |
| <application_data> | |  |  |  |  |  |  |  |  |
| <service_mode></service_mode> | | | |  |  |  |  |  |  |
| <query_desc>AdvancedSearch</query_desc> | | | | |  |  |  |  |  |
| <query_type>com.thomson.ts.framework.query.IGeneralQuery</query_type> | | | | | | | |  |  |
| <preparsed><![CDATA[TS=（femoral neck fracture or Femoral Neck Fracture or Femur Neck Fractures or Femur Neck Fracture ） ]]></preparsed> | | | | | | | | | |
| <molecule><![CDATA[]]></molecule> | | | |  |  |  |  |  |  |
| <frag_codes><![CDATA[]]></frag_codes> | | | |  |  |  |  |  |  |
| <queryType>com.thomson.ts.framework.query.IGeneralQuery</queryType> | | | | | | | |  |  |
| <query_syntax>3.3</query_syntax> | | | |  |  |  |  |  |  |
| <oldQuerySyntax></oldQuerySyntax> | | | |  |  |  |  |  |  |
| <search_mode>AdvancedSearch</search_mode> | | | | |  |  |  |  |  |
| <colName></colName> | | |  |  |  |  |  |  |  |
| <unparsedSearchInput><![CDATA[TS=（femoral neck fracture or Femoral Neck Fracture or Femur Neck Fractures or Femur Neck Fracture ） ]]></unparsedSearchInput> | | | | | | | | | |
| <parsedSearchInput><![CDATA[]]></parsedSearchInput> | | | | | |  |  |  |  |
| <unparsedSearchNoLimits><![CDATA[TS=（femoral neck fracture or Femoral Neck Fracture or Femur Neck Fractures or Femur Neck Fracture ） ]]></unparsedSearchNoLimits> | | | | | | | | | |
| <subtype></subtype> | | |  |  |  |  |  |  |  |
| <options></options> | | |  |  |  |  |  |  |  |
| <user_query_string_xml><Field><![CDATA[TS=（femoral neck fracture or Femoral Neck Fracture or Femur Neck Fractures or Femur Neck Fracture ）]]></Field></user_query_string_xml> | | | | | | | | | |
| <ss_lemmatization><![CDATA[On]]></ss_lemmatization> | | | | | |  |  |  |  |
| <ss_spellchecking><![CDATA[Suggest]]></ss_spellchecking> | | | | | |  |  |  |  |
| <ss_query_language><![CDATA[auto]]></ss_query_language> | | | | | |  |  |  |  |
| <isOneClickDaisyAuthor></isOneClickDaisyAuthor> | | | | |  |  |  |  |  |
| <OneClickDaisyAuthorName></OneClickDaisyAuthorName> | | | | | |  |  |  |  |
| <relatedMetaData></relatedMetaData> | | | |  |  |  |  |  |  |
| <parentUT></parentUT> | | |  |  |  |  |  |  |  |
| </application_data> | |  |  |  |  |  |  |  |  |
| </set> |  |  |  |  |  |  |  |  |  |
| <set> |  |  |  |  |  |  |  |  |  |
| <set_number>4</set_number> | | |  |  |  |  |  |  |  |
| <field_id></field_id> | |  |  |  |  |  |  |  |  |
| <tns_id></tns_id> | |  |  |  |  |  |  |  |  |
| <parsed_query_string><![CDATA[]]></parsed_query_string> | | | | | |  |  |  |  |
| <user_query_string><![CDATA[TS=（ Femur Head Necrosis or Femur Head Necroses or Head Necrosis | Femur or Necrosis | Femur Head or Aseptic Necrosis of Femur Head or Necrosis | Aseptic | of Femur Head or Ischemic Necrosis Of Femoral Head or Femoral Head | Avascular Necrosis Of or Avascular Necrosis Of Femoral Head | Primary or Avascular Necrosis of Femur Head ）]]></user_query_string> | | | |
| <user_timespan_string><![CDATA[1962-2019]]></user_timespan_string> | | | | | | |  |  |  |
| <parsed_dblimits_string><![CDATA[WOS.IC WOS.SCI WOS.CCR KJD.KJD MEDLINE.MEDLINE RSCI.RSCI SCIELO.SCIELO]]></parsed_dblimits_string> | | | | | | | | | |
| <user_editions_string><![CDATA[ WOS | KJD | MEDLINE | RSCI | SCIELO]]></user_editions_string> | | | |  |  |
| <user_limits_string><![CDATA[]]></user_limits_string> | | | | | |  |  |  |  |
| <refinedBy><![CDATA[]]></refinedBy> | | | |  |  |  |  |  |  |
| <analysisHistory></analysisHistory> | | | |  |  |  |  |  |  |
| <application_data> | |  |  |  |  |  |  |  |  |
| <service_mode></service_mode> | | | |  |  |  |  |  |  |
| <query_desc>AdvancedSearch</query_desc> | | | | |  |  |  |  |  |
| <query_type>com.thomson.ts.framework.query.IGeneralQuery</query_type> | | | | | | | |  |  |
| <preparsed><![CDATA[TS=（ Femur Head Necrosis or Femur Head Necroses or Head Necrosis | Femur or Necrosis | Femur Head or Aseptic Necrosis of Femur Head or Necrosis | Aseptic | of Femur Head or Ischemic Necrosis Of Femoral Head or Femoral Head | Avascular Necrosis Of or Avascular Necrosis Of Femoral Head | Primary or Avascular Necrosis of Femur Head ） ]]></preparsed> | | | |
| <molecule><![CDATA[]]></molecule> | | | |  |  |  |  |  |  |
| <frag_codes><![CDATA[]]></frag_codes> | | | |  |  |  |  |  |  |
| <queryType>com.thomson.ts.framework.query.IGeneralQuery</queryType> | | | | | | | |  |  |
| <query_syntax>3.3</query_syntax> | | | |  |  |  |  |  |  |
| <oldQuerySyntax></oldQuerySyntax> | | | |  |  |  |  |  |  |
| <search_mode>AdvancedSearch</search_mode> | | | | |  |  |  |  |  |
| <colName></colName> | | |  |  |  |  |  |  |  |
| <unparsedSearchInput><![CDATA[TS=（ Femur Head Necrosis or Femur Head Necroses or Head Necrosis | Femur or Necrosis | Femur Head or Aseptic Necrosis of Femur Head or Necrosis | Aseptic | of Femur Head or Ischemic Necrosis Of Femoral Head or Femoral Head | Avascular Necrosis Of or Avascular Necrosis Of Femoral Head | Primary or Avascular Necrosis of Femur Head ） ]]></unparsedSearchInput> | | | |
| <parsedSearchInput><![CDATA[]]></parsedSearchInput> | | | | | |  |  |  |  |
| <unparsedSearchNoLimits><![CDATA[TS=（ Femur Head Necrosis or Femur Head Necroses or Head Necrosis | Femur or Necrosis | Femur Head or Aseptic Necrosis of Femur Head or Necrosis | Aseptic | of Femur Head or Ischemic Necrosis Of Femoral Head or Femoral Head | Avascular Necrosis Of or Avascular Necrosis Of Femoral Head | Primary or Avascular Necrosis of Femur Head ） ]]></unparsedSearchNoLimits> | | | |
| <subtype></subtype> | | |  |  |  |  |  |  |  |
| <options></options> | | |  |  |  |  |  |  |  |
| <user_query_string_xml><Field><![CDATA[TS=（ Femur Head Necrosis or Femur Head Necroses or Head Necrosis | Femur or Necrosis | Femur Head or Aseptic Necrosis of Femur Head or Necrosis | Aseptic | of Femur Head or Ischemic Necrosis Of Femoral Head or Femoral Head | Avascular Necrosis Of or Avascular Necrosis Of Femoral Head | Primary or Avascular Necrosis of Femur Head ）]]></Field></user_query_string_xml> | | | |
| <ss_lemmatization><![CDATA[On]]></ss_lemmatization> | | | | | |  |  |  |  |
| <ss_spellchecking><![CDATA[Suggest]]></ss_spellchecking> | | | | | |  |  |  |  |
| <ss_query_language><![CDATA[auto]]></ss_query_language> | | | | | |  |  |  |  |
| <isOneClickDaisyAuthor></isOneClickDaisyAuthor> | | | | |  |  |  |  |  |
| <OneClickDaisyAuthorName></OneClickDaisyAuthorName> | | | | | |  |  |  |  |
| <relatedMetaData></relatedMetaData> | | | |  |  |  |  |  |  |
| <parentUT></parentUT> | | |  |  |  |  |  |  |  |
| </application_data> | |  |  |  |  |  |  |  |  |
| </set> |  |  |  |  |  |  |  |  |  |
| <set> |  |  |  |  |  |  |  |  |  |
| <set_number>5</set_number> | | |  |  |  |  |  |  |  |
| <field_id></field_id> | |  |  |  |  |  |  |  |  |
| <tns_id>UA</tns_id> | | |  |  |  |  |  |  |  |
| <parsed_query_string><![CDATA[]]></parsed_query_string> | | | | | |  |  |  |  |
| <user_query_string><![CDATA[#2 OR #1]]></user_query_string> | | | | | | |  |  |  |
| <user_timespan_string><![CDATA[1962-2019]]></user_timespan_string> | | | | | | |  |  |  |
| <parsed_dblimits_string><![CDATA[WOS.IC WOS.SCI WOS.CCR KJD.KJD MEDLINE.MEDLINE RSCI.RSCI SCIELO.SCIELO]]></parsed_dblimits_string> | | | | | | | | | |
| <user_editions_string><![CDATA[ WOS | KJD | MEDLINE | RSCI | SCIELO]]></user_editions_string> | | | |  |  |
| <user_limits_string><![CDATA[]]></user_limits_string> | | | | | |  |  |  |  |
| <refinedBy><![CDATA[]]></refinedBy> | | | |  |  |  |  |  |  |
| <analysisHistory></analysisHistory> | | | |  |  |  |  |  |  |
| <application_data> | |  |  |  |  |  |  |  |  |
| <service_mode></service_mode> | | | |  |  |  |  |  |  |
| <query_desc>CombineSearches</query_desc> | | | | |  |  |  |  |  |
| <query_type>com.thomson.ts.framework.query.IGeneralQuery</query_type> | | | | | | | |  |  |
| <preparsed><![CDATA[#2 OR #1]]></preparsed> | | | | |  |  |  |  |  |
| <molecule><![CDATA[]]></molecule> | | | |  |  |  |  |  |  |
| <frag_codes><![CDATA[]]></frag_codes> | | | |  |  |  |  |  |  |
| <queryType>com.thomson.ts.framework.query.IGeneralQuery</queryType> | | | | | | | |  |  |
| <query_syntax>3.3</query_syntax> | | | |  |  |  |  |  |  |
| <oldQuerySyntax></oldQuerySyntax> | | | |  |  |  |  |  |  |
| <search_mode>CombineSearches</search_mode> | | | | |  |  |  |  |  |
| <colName></colName> | | |  |  |  |  |  |  |  |
| <unparsedSearchInput><![CDATA[#2 OR #1]]></unparsedSearchInput> | | | | | | |  |  |  |
| <parsedSearchInput><![CDATA[]]></parsedSearchInput> | | | | | |  |  |  |  |
| <unparsedSearchNoLimits><![CDATA[#2 OR #1]]></unparsedSearchNoLimits> | | | | | | | |  |  |
| <subtype></subtype> | | |  |  |  |  |  |  |  |
| <options></options> | | |  |  |  |  |  |  |  |
| <user_query_string_xml>#2 OR #1</user_query_string_xml> | | | | | |  |  |  |  |
| <ss_lemmatization><![CDATA[On]]></ss_lemmatization> | | | | | |  |  |  |  |
| <ss_spellchecking><![CDATA[Suggest]]></ss_spellchecking> | | | | | |  |  |  |  |
| <ss_query_language><![CDATA[auto]]></ss_query_language> | | | | | |  |  |  |  |
| <isOneClickDaisyAuthor></isOneClickDaisyAuthor> | | | | |  |  |  |  |  |
| <OneClickDaisyAuthorName></OneClickDaisyAuthorName> | | | | | |  |  |  |  |
| <relatedMetaData></relatedMetaData> | | | |  |  |  |  |  |  |
| <parentUT></parentUT> | | |  |  |  |  |  |  |  |
| </application_data> | |  |  |  |  |  |  |  |  |
| </set> |  |  |  |  |  |  |  |  |  |
| <set> |  |  |  |  |  |  |  |  |  |
| <set_number>6</set_number> | | |  |  |  |  |  |  |  |
| <field_id></field_id> | |  |  |  |  |  |  |  |  |
| <tns_id>UA</tns_id> | | |  |  |  |  |  |  |  |
| <parsed_query_string><![CDATA[]]></parsed_query_string> | | | | | |  |  |  |  |
| <user_query_string><![CDATA[#5 AND #4 AND #3]]></user_query_string> | | | | | | |  |  |  |
| <user_timespan_string><![CDATA[1962-2019]]></user_timespan_string> | | | | | | |  |  |  |
| <parsed_dblimits_string><![CDATA[WOS.IC WOS.SCI WOS.CCR KJD.KJD MEDLINE.MEDLINE RSCI.RSCI SCIELO.SCIELO]]></parsed_dblimits_string> | | | | | | | | | |
| <user_editions_string><![CDATA[ WOS | KJD | MEDLINE | RSCI | SCIELO]]></user_editions_string> | | | |  |  |
| <user_limits_string><![CDATA[]]></user_limits_string> | | | | | |  |  |  |  |
| <refinedBy><![CDATA[]]></refinedBy> | | | |  |  |  |  |  |  |
| <analysisHistory></analysisHistory> | | | |  |  |  |  |  |  |
| <application_data> | |  |  |  |  |  |  |  |  |
| <service_mode></service_mode> | | | |  |  |  |  |  |  |
| <query_desc>CombineSearches</query_desc> | | | | |  |  |  |  |  |
| <query_type>com.thomson.ts.framework.query.IGeneralQuery</query_type> | | | | | | | |  |  |
| <preparsed><![CDATA[#5 AND #4 AND #3]]></preparsed> | | | | | |  |  |  |  |
| <molecule><![CDATA[]]></molecule> | | | |  |  |  |  |  |  |
| <frag_codes><![CDATA[]]></frag_codes> | | | |  |  |  |  |  |  |
| <queryType>com.thomson.ts.framework.query.IGeneralQuery</queryType> | | | | | | | |  |  |
| <query_syntax>3.3</query_syntax> | | | |  |  |  |  |  |  |
| <oldQuerySyntax></oldQuerySyntax> | | | |  |  |  |  |  |  |
| <search_mode>CombineSearches</search_mode> | | | | |  |  |  |  |  |
| <colName></colName> | | |  |  |  |  |  |  |  |
| <unparsedSearchInput><![CDATA[#5 AND #4 AND #3]]></unparsedSearchInput> | | | | | | | |  |  |
| <parsedSearchInput><![CDATA[]]></parsedSearchInput> | | | | | |  |  |  |  |
| <unparsedSearchNoLimits><![CDATA[#5 AND #4 AND #3]]></unparsedSearchNoLimits> | | | | | | | | |  |
| <subtype></subtype> | | |  |  |  |  |  |  |  |
| <options></options> | | |  |  |  |  |  |  |  |
| <user_query_string_xml>#5 AND #4 AND #3</user_query_string_xml> | | | | | | |  |  |  |
| <ss_lemmatization><![CDATA[On]]></ss_lemmatization> | | | | | |  |  |  |  |
| <ss_spellchecking><![CDATA[Suggest]]></ss_spellchecking> | | | | | |  |  |  |  |
| <ss_query_language><![CDATA[auto]]></ss_query_language> | | | | | |  |  |  |  |
| <isOneClickDaisyAuthor></isOneClickDaisyAuthor> | | | | |  |  |  |  |  |
| <OneClickDaisyAuthorName></OneClickDaisyAuthorName> | | | | | |  |  |  |  |
| <relatedMetaData></relatedMetaData> | | | |  |  |  |  |  |  |
| <parentUT></parentUT> | | |  |  |  |  |  |  |  |
| </application_data> | |  |  |  |  |  |  |  |  |
| </set> |  |  |  |  |  |  |  |  |  |
| </sets> |  |  |  |  |  |  |  |  |  |
| </search> | |  |  |  |  |  |  |  |  |
