# Supplementary material for: The clinical and radiographic characteristics of avascular necrosis after pediatric femoral neck fracture: a systematic review and retrospective study of 115 patients
Source: J Orthop Surg Res. 2020 Nov 11;15:520. doi: 10.1186/s13018-020-02037-2 (PMC7661253; doi:10.1186/s13018-020-02037-2)
Supplement: Supplementary file 2 — Additional file 2. IHE’s quality appraisal checklist for assessing case-series studies. [file 13018_2020_2037_MOESM2_ESM.docx]

IHE’s quality appraisal checklist for assessing case-series studies

Table A.1. Quality appraisal checklist for case-series studies and guidance

Criterion and guidance for rating each criterion

Study objective

1. Was the hypothesis/aim/objective of the study clearly stated?

Yes: The hypothesis/aim/objective of the study was clearly reported (includes patients, intervention, and outcome). Partial: Only one or two components (patients, intervention, or outcome) were included. No: The hypothesis/aim/objective was not reported.

Study design

1. Was the study conducted prospectively?

Yes: It was clearly stated that the study was conducted prospectively.

Unclear: Unclear or no information was provided.

No: The study clearly stated it was a retrospective study.

1. Were the cases collected in more than one center?

Yes: Cases were collected in more than one center (multicenter study).

Unclear: Unclear where the patients came from. No: Cases were collected from one center.

1. Were patients recruited consecutively?

Yes: There was a clear statement or it was clear from the context that the patients were recruited consecutively; or the study stated that all eligible patients were recruited.

Unclear: No information was provided about the method used to recruit patients in the study.

No: The study clearly stated that patients were not recruited consecutively; or the patients were recruited based on other criteria such as access to intervention determined by the distance or availability of resources.

Study population

1. Were the characteristics of the patients included in the study described?

Yes: All the most relevant characteristics of the patients were reported (eg, number, age, gender, ethnicity, severity of disease/condition, comorbidity, or etiology).

Partial: Some, but not all, of the most relevant characteristics were reported.

No: Only the number of patients was reported.

Note: Assessor(s) should decide which aspects are important before using the checklist.

1. Were the eligibility criteria (ie, inclusion and exclusion criteria) for entry into the study clearly stated?

Yes: Both inclusion and exclusion criteria were reported.

Partial: Either the inclusion or exclusion criteria were reported.

No: Neither inclusion nor exclusion criteria were reported.

Note: Assessor(s) should decide which aspects are important before using the checklist.

1. Did patients enter the study at a similar point in the disease?

Yes: It was clear from the baseline data presented in the study (eg, tables of patients’ characteristics) that most (at least 80%) patients entered the study at a similar point in terms of the duration and severity of the disease/condition and the presence of comorbidities/ complications.

Unclear: There was no baseline information on patients’ characteristics to make a judgment.

No: There was a wide range in the severity of the disease/condition and comorbidities/complications in patients at baseline.

Note: Assessor(s) should decide which aspects are important before using the checklist. It might be useful to discuss with specialists to determine the most important aspects that should be considered.

Intervention and cointervention

1. Was the intervention of interest clearly described?

Yes: All the most relevant characteristics of the intervention were reported (eg, dosage, frequency or duration of intervention, administration methods, technical parameters, or characteristics of a device).

Partial: Some, but not all, of the most relevant characteristics were reported.

No: Only the name of the intervention was reported.

Note: Assessor(s) should decide which aspects are important before using the checklist.

1. Were additional interventions (cointerventions) clearly described?

Yes: All the most relevant characteristics of the cointervention(s) were reported (eg, different type, dosage, frequency of administration, or duration); or the study clearly stated that a cointervention was not administered for clinical reasons.

Partial: Some, but not all, of the most relevant characteristics of the cointervention were reported.

No: No information about cointervention(s) was provided; or only the name(s) of the cointervention(s) were mentioned. Note: Assessor(s) should decide which aspects are important before using the checklist.

Outcome measures

10.Were relevant outcome measures established a priori?

Yes: All relevant outcome measures were stated in the introduction or methods section.

Partial: Some, but not all, of the relevant outcome measures were stated in the introduction or method section. No: None of the relevant outcome measures were stated in the introduction or method section.

(Continued)

B. Guo et al. / Journal of Clinical Epidemiology 69 (2016) 199 207.e2

Table A.1. Continued

Criterion and guidance for rating each criterion

11.Were outcome assessors blinded to the intervention that patients received?

Yes: The relevant outcomes were assessed by individuals who were not aware of the intervention. Answer yes when blinding is not applicable or is unnecessary (eg, mortality).

Unclear: The study did not report whether the outcome assessors were aware of the intervention.

No: It was clearly stated or obvious from the context that the relevant outcomes were analyzed by individuals who were aware of the intervention provided to patients.

12.Were the relevant outcomes measured using appropriate objective/subjective methods?

Yes: All relevant outcomes were measured with appropriate methods. These measures can be objective (eg, gold standard tests or standardized clinical tests), subjective (eg, self-administered questionnaires, standardized forms, or patient symptoms interview forms), or both.

Partial: Some, but not all, relevant outcomes were measured with appropriate methods.

No: The methods used to measure the relevant outcomes were inappropriate.

Note: Assessor(s) should decide which methods are appropriate before using the checklist.

13.Were the relevant outcome measures made before and after the intervention?

Yes: The relevant outcome measures were made before and after intervention; or the baseline measurements were not possible (eg, death). Unclear: The study did not report when the outcome measures were made. No: The outcome measures were only made after intervention. Statistical analysis

14.Were the statistical tests used to assess the relevant outcomes appropriate?

Yes: The statistical tests were used appropriately (eg, parametric test for normally distributed population vs. nonparametric test for nonGaussian population). Answer ‘‘yes’’ if no statistical analysis was performed and reasons for this were stated. Unclear: The statistical tests were not described in the methods section of the study.

No: The statistical tests used were inappropriate.

Note: Assessor(s) should decide which statistical tests are appropriate before using the checklist. Seek expert assistance if necessary.

Results and conclusions

15.Was follow-up long enough for important events and outcomes to occur?

Yes: It was clear from the information provided that the follow-up period was long enough for most (at least 80%) patients, to allow for important events and outcomes (eg, changes in clinical status, adverse events) to occur.

Unclear: The length of follow-up was not clearly reported.

No: It is clear from the information provided that the follow-up period was not long enough to allow for important events and outcomes to occur.

Note: Assessor(s) should define the appropriate duration of follow-up for each outcome of interest (eg, short-term and long-term adverse events).

16.Were losses to follow-up reported?

Yes: The number or proportion of patients lost to follow-up was clearly reported; the authors reported outcome results on all patients initially included; or the number lost to follow-up can be subtracted from the number of patients enrolled and the number of patients included in the final analysis.

Unclear: There was a discrepancy between the number or proportion of patients reported in tables, figures, and text. No: The number or proportion of patients lost to follow-up was not reported.

17.Did the study provided estimates of random variability in the data analysis of relevant outcomes?

Yes: The estimates of the random variability (eg, standard error, standard deviation, confidence interval for normally distributed data or range, and interquartile range for nonnormally distributed data) were reported for all the relevant outcomes or could be calculated from the raw data presented in the study.

Partial: The estimates of the random variability were reported for some, but not all, of the relevant outcomes.

No: The estimates of the random variability were not reported for any of the relevant outcomes.

18.Were the adverse events reported?

Yes: The undesirable or unwanted events during the study period or within a prespecified time period were reported; or the absence of adverse event(s) was mentioned in the study.

Partial: Some, but not all, important adverse events were reported.

No: There was no statement about the presence or absence of adverse events.

Note: Assessor(s) should decide which adverse events are most important. Seek clinical expert assistance if necessary.

19.Were the conclusions of the study supported by the results?

Yes: The conclusions of the study were supported by the evidence presented in the results and discussion sections.

Unclear: Unclear conclusion statement that makes it difficult to link the presented evidence to conclusions. No: The conclusions were not supported by the evidence presented in the results and discussion sections.

Competing interests and sources of support

20.Were both competing interests and sources of support for the study reported?

Yes: Both competing interests and sources of support (financial or other) received for the study were reported; or the absence of any competing interest and source of support was acknowledged.

Partial: Either the competing interest or source of support was reported. No: Neither competing interests nor sources of support were reported.

Note: Assessor(s) may decide to remove from the checklist the criteria that are not applicable to their project.

| **Study objective** | Yes | Partial | unclear | No |
| --- | --- | --- | --- | --- |
| 1.Was the hypothesis/aim/objective of the study clearly stated? Study design | 1 |  |  |  |
| **Study design** |  |  |  |  |
| 2.Was the study conducted prospectively? |  |  |  | 1 |
| 3.Were the cases collected in more than one center? |  |  |  | 1 |
| 4.Were patients recruited consecutively? |  |  | 1 |  |
| **Study population** |  |  |  |  |
| 5. Were the characteristics of the patients included in the study described? |  | 1 |  |  |
| 6. Were the eligibility criteria (i.e. inclusion and exclusion criteria) for entry into the study clearly stated? | 1 |  |  |  |
| 7. Did patients enter the study at a similar point in the disease? | 1 |  |  |  |
| **Intervention and cointervention** |  |  |  |  |
| 8.Was the intervention of interest clearly described? | 1 |  |  |  |
| 9.Were additional interventions (cointerventions) clearly described? | 1 |  |  |  |
| **Outcome measures** |  |  |  |  |
| 10.Were relevant outcome measures established a priori? | 1 |  |  |  |
| 11.Were outcome assessors blinded to the intervention that patients received? |  |  | 1 |  |
| 12.Were the relevant outcomes measured using appropriate objective/subjective methods? | 1 |  |  |  |
| 13.Were the relevant outcome measures made before and after the intervention? |  |  |  | 1 |
| **Statistical analysis** |  |  |  |  |
| 14.Were the statistical tests used to assess the relevant outcomes appropriate? | 1 |  |  |  |
| **Results and conclusions** |  |  |  |  |
| 15.Was follow-up long enough for important events and outcomes to occur? | 1 |  |  |  |
| 16.Were losses to follow-up reported? | 1 |  |  |  |
| 17.Did the study provided estimates of random variability in the data analysis of relevant outcomes? |  |  | 1 |  |
| 18.Were the adverse events reported? | 1 |  |  |  |
| 19.Were the conclusions of the study supported by the results? | 1 |  |  |  |
| **Competing interests and sources of support** |  |  |  |  |
| 20.Were both competing interests and sources of support for the study reported? |  |  |  | 1 |
| Total score | 12 | 1 |  |  |

1.Stone

2015

2.Panigrahi

2015

| **Study objective** | Yes | Partial | unclear | No |
| --- | --- | --- | --- | --- |
| 1.Was the hypothesis/aim/objective of the study clearly stated? Study design | 1 |  |  |  |
| **Study design** |  |  |  |  |
| 2.Was the study conducted prospectively? | 1 |  |  |  |
| 3.Were the cases collected in more than one center? | 1 |  |  |  |
| 4.Were patients recruited consecutively? |  |  | 1 |  |
| **Study population** |  |  |  |  |
| 5. Were the characteristics of the patients included in the study described? |  | 1 |  |  |
| 6. Were the eligibility criteria (i.e. inclusion and exclusion criteria) for entry into the study clearly stated? | 1 |  |  |  |
| 7. Did patients enter the study at a similar point in the disease? | 1 |  |  |  |
| **Intervention and cointervention** |  |  |  |  |
| 8.Was the intervention of interest clearly described? |  | 1 |  |  |
| 9.Were additional interventions (cointerventions) clearly described? |  | 1 |  |  |
| **Outcome measures** |  |  |  |  |
| 10.Were relevant outcome measures established a priori? | 1 |  |  |  |
| 11.Were outcome assessors blinded to the intervention that patients received? |  |  | 1 |  |
| 12.Were the relevant outcomes measured using appropriate objective/subjective methods? | 1 |  |  |  |
| 13.Were the relevant outcome measures made before and after the intervention? |  |  |  | 1 |
| **Statistical analysis** |  |  |  |  |
| 14.Were the statistical tests used to assess the relevant outcomes appropriate? |  |  | 1 |  |
| **Results and conclusions** |  |  |  |  |
| 15.Was follow-up long enough for important events and outcomes to occur? | 1 |  |  |  |
| 16.Were losses to follow-up reported? | 1 |  |  |  |
| 17.Did the study provided estimates of random variability in the data analysis of relevant outcomes? |  |  | 1 |  |
| 18.Were the adverse events reported? | 1 |  |  |  |
| 19.Were the conclusions of the study supported by the results? | 1 |  |  |  |
| **Competing interests and sources of support** |  |  |  |  |
| 20.Were both competing interests and sources of support for the study reported? |  |  |  | 1 |
| Total score | 11 | 2 |  |  |

3.Bojan

2015

| **Study objective** | Yes | Partial | unclear | No |
| --- | --- | --- | --- | --- |
| 1.Was the hypothesis/aim/objective of the study clearly stated? Study design | 1 |  |  |  |
| **Study design** |  |  |  |  |
| 2.Was the study conducted prospectively? |  |  |  | 1 |
| 3.Were the cases collected in more than one center? |  |  |  | 1 |
| 4.Were patients recruited consecutively? |  |  | 1 |  |
| **Study population** |  |  |  |  |
| 5. Were the characteristics of the patients included in the study described? |  | 1 |  |  |
| 6. Were the eligibility criteria (i.e. inclusion and exclusion criteria) for entry into the study clearly stated? |  | 1 |  |  |
| 7. Did patients enter the study at a similar point in the disease? | 1 |  |  |  |
| **Intervention and cointervention** |  |  |  |  |
| 8.Was the intervention of interest clearly described? |  | 1 |  |  |
| 9.Were additional interventions (cointerventions) clearly described? |  |  |  | 1 |
| **Outcome measures** |  |  |  |  |
| 10.Were relevant outcome measures established a priori? | 1 |  |  |  |
| 11.Were outcome assessors blinded to the intervention that patients received? |  |  | 1 |  |
| 12.Were the relevant outcomes measured using appropriate objective/subjective methods? | 1 |  |  |  |
| 13.Were the relevant outcome measures made before and after the intervention? |  |  |  | 1 |
| **Statistical analysis** |  |  |  |  |
| 14.Were the statistical tests used to assess the relevant outcomes appropriate? | 1 |  |  |  |
| **Results and conclusions** |  |  |  |  |
| 15.Was follow-up long enough for important events and outcomes to occur? | 1 |  |  |  |
| 16.Were losses to follow-up reported? | 1 |  |  |  |
| 17.Did the study provided estimates of random variability in the data analysis of relevant outcomes? |  |  | 1 |  |
| 18.Were the adverse events reported? | 1 |  |  |  |
| 19.Were the conclusions of the study supported by the results? |  | 1 |  |  |
| **Competing interests and sources of support** |  |  |  |  |
| 20.Were both competing interests and sources of support for the study reported? |  | 1 |  |  |
| Total score | 8 | 5 |  |  |

4.Stefan

2011

| **Study objective** | Yes | Partial | unclear | No |
| --- | --- | --- | --- | --- |
| 1.Was the hypothesis/aim/objective of the study clearly stated? Study design | 1 |  |  |  |
| **Study design** |  |  |  |  |
| 2.Was the study conducted prospectively? |  |  |  | 1 |
| 3.Were the cases collected in more than one center? |  |  |  | 1 |
| 4.Were patients recruited consecutively? | 1 |  |  |  |
| **Study population** |  |  |  |  |
| 5. Were the characteristics of the patients included in the study described? |  | 1 |  |  |
| 6. Were the eligibility criteria (i.e. inclusion and exclusion criteria) for entry into the study clearly stated? | 1 |  |  |  |
| 7. Did patients enter the study at a similar point in the disease? | 1 |  |  |  |
| **Intervention and cointervention** |  |  |  |  |
| 8.Was the intervention of interest clearly described? |  |  |  | 1 |
| 9.Were additional interventions (cointerventions) clearly described? |  |  |  | 1 |
| **Outcome measures** |  |  |  |  |
| 10.Were relevant outcome measures established a priori? | 1 |  |  |  |
| 11.Were outcome assessors blinded to the intervention that patients received? |  |  | 1 |  |
| 12.Were the relevant outcomes measured using appropriate objective/subjective methods? | 1 |  |  |  |
| 13.Were the relevant outcome measures made before and after the intervention? |  |  | 1 |  |
| **Statistical analysis** |  |  |  |  |
| 14.Were the statistical tests used to assess the relevant outcomes appropriate? |  |  | 1 |  |
| **Results and conclusions** |  |  |  |  |
| 15.Was follow-up long enough for important events and outcomes to occur? | 1 |  |  |  |
| 16.Were losses to follow-up reported? | 1 |  |  |  |
| 17.Did the study provided estimates of random variability in the data analysis of relevant outcomes? |  |  | 1 |  |
| 18.Were the adverse events reported? | 1 |  |  |  |
| 19.Were the conclusions of the study supported by the results? |  |  | 1 |  |
| **Competing interests and sources of support** |  |  |  |  |
| 20.Were both competing interests and sources of support for the study reported? |  | 1 |  |  |
| Total score | 9 | 2 |  |  |

5.Kamal Bali

2011

| **Study objective** | Yes | Partial | unclear | No |
| --- | --- | --- | --- | --- |
| 1.Was the hypothesis/aim/objective of the study clearly stated? Study design | 1 |  |  |  |
| **Study design** |  |  |  |  |
| 2.Was the study conducted prospectively? |  |  |  | 1 |
| 3.Were the cases collected in more than one center? |  |  |  | 1 |
| 4.Were patients recruited consecutively? |  |  | 1 |  |
| **Study population** |  |  |  |  |
| 5. Were the characteristics of the patients included in the study described? | 1 |  |  |  |
| 6. Were the eligibility criteria (i.e. inclusion and exclusion criteria) for entry into the study clearly stated? |  |  |  | 1 |
| 7. Did patients enter the study at a similar point in the disease? | 1 |  |  |  |
| **Intervention and cointervention** |  |  |  |  |
| 8.Was the intervention of interest clearly described? |  |  |  | 1 |
| 9.Were additional interventions (cointerventions) clearly described? |  |  |  | 1 |
| **Outcome measures** |  |  |  |  |
| 10.Were relevant outcome measures established a priori? | 1 |  |  |  |
| 11.Were outcome assessors blinded to the intervention that patients received? |  |  | 1 |  |
| 12.Were the relevant outcomes measured using appropriate objective/subjective methods? | 1 |  |  |  |
| 13.Were the relevant outcome measures made before and after the intervention? |  |  |  | 1 |
| **Statistical analysis** |  |  |  |  |
| 14.Were the statistical tests used to assess the relevant outcomes appropriate? |  |  | 1 |  |
| **Results and conclusions** |  |  |  |  |
| 15.Was follow-up long enough for important events and outcomes to occur? | 1 |  |  |  |
| 16.Were losses to follow-up reported? | 1 |  |  |  |
| 17.Did the study provided estimates of random variability in the data analysis of relevant outcomes? |  |  | 1 |  |
| 18.Were the adverse events reported? | 1 |  |  |  |
| 19.Were the conclusions of the study supported by the results? |  |  | 1 |  |
| **Competing interests and sources of support** |  |  |  |  |
| 20.Were both competing interests and sources of support for the study reported? |  | 1 |  |  |
| Total score | 8 | 1 |  |  |

6. Mohammed

2009

| **Study objective** | Yes | Partial | unclear | No |
| --- | --- | --- | --- | --- |
| 1.Was the hypothesis/aim/objective of the study clearly stated? Study design | 1 |  |  |  |
| **Study design** |  |  |  |  |
| 2.Was the study conducted prospectively? |  |  |  | 1 |
| 3.Were the cases collected in more than one center? |  |  |  | 1 |
| 4.Were patients recruited consecutively? |  |  | 1 |  |
| **Study population** |  |  |  |  |
| 5. Were the characteristics of the patients included in the study described? |  | 1 |  |  |
| 6. Were the eligibility criteria (i.e. inclusion and exclusion criteria) for entry into the study clearly stated? |  |  |  | 1 |
| 7. Did patients enter the study at a similar point in the disease? | 1 |  |  |  |
| **Intervention and cointervention** |  |  |  |  |
| 8.Was the intervention of interest clearly described? |  |  |  | 1 |
| 9.Were additional interventions (cointerventions) clearly described? |  |  |  | 1 |
| **Outcome measures** |  |  |  |  |
| 10.Were relevant outcome measures established a priori? | 1 |  |  |  |
| 11.Were outcome assessors blinded to the intervention that patients received? |  |  | 1 |  |
| 12.Were the relevant outcomes measured using appropriate objective/subjective methods? |  |  | 1 |  |
| 13.Were the relevant outcome measures made before and after the intervention? |  |  |  | 1 |
| **Statistical analysis** |  |  |  |  |
| 14.Were the statistical tests used to assess the relevant outcomes appropriate? | 1 |  |  |  |
| **Results and conclusions** |  |  |  |  |
| 15.Was follow-up long enough for important events and outcomes to occur? | 1 |  |  |  |
| 16.Were losses to follow-up reported? | 1 |  |  |  |
| 17.Did the study provided estimates of random variability in the data analysis of relevant outcomes? |  |  | 1 |  |
| 18.Were the adverse events reported? | 1 |  |  |  |
| 19.Were the conclusions of the study supported by the results? |  | 1 |  |  |
| **Competing interests and sources of support** |  |  |  |  |
| 20.Were both competing interests and sources of support for the study reported? |  |  |  | 1 |
| Total score | 7 | 2 |  |  |

7.Ulukan

2009

| **Study objective** | Yes | Partial | unclear | No |
| --- | --- | --- | --- | --- |
| 1.Was the hypothesis/aim/objective of the study clearly stated? Study design | 1 |  |  |  |
| **Study design** |  |  |  |  |
| 2.Was the study conducted prospectively? |  |  |  | 1 |
| 3.Were the cases collected in more than one center? |  |  |  | 1 |
| 4.Were patients recruited consecutively? | 1 |  |  |  |
| **Study population** |  |  |  |  |
| 5. Were the characteristics of the patients included in the study described? |  | 1 |  |  |
| 6. Were the eligibility criteria (i.e. inclusion and exclusion criteria) for entry into the study clearly stated? |  |  |  | 1 |
| 7. Did patients enter the study at a similar point in the disease? | 1 |  |  |  |
| **Intervention and cointervention** |  |  |  |  |
| 8.Was the intervention of interest clearly described? | 1 |  |  |  |
| 9.Were additional interventions (cointerventions) clearly described? |  | 1 |  |  |
| **Outcome measures** |  |  |  |  |
| 10.Were relevant outcome measures established a priori? | 1 |  |  |  |
| 11.Were outcome assessors blinded to the intervention that patients received? |  |  | 1 |  |
| 12.Were the relevant outcomes measured using appropriate objective/subjective methods? | 1 |  |  |  |
| 13.Were the relevant outcome measures made before and after the intervention? |  |  |  | 1 |
| **Statistical analysis** |  |  |  |  |
| 14.Were the statistical tests used to assess the relevant outcomes appropriate? | 1 |  |  |  |
| **Results and conclusions** |  |  |  |  |
| 15.Was follow-up long enough for important events and outcomes to occur? | 1 |  |  |  |
| 16.Were losses to follow-up reported? | 1 |  |  |  |
| 17.Did the study provided estimates of random variability in the data analysis of relevant outcomes? |  |  | 1 |  |
| 18.Were the adverse events reported? | 1 |  |  |  |
| 19.Were the conclusions of the study supported by the results? | 1 |  |  |  |
| **Competing interests and sources of support** |  |  |  |  |
| 20.Were both competing interests and sources of support for the study reported? |  | 1 |  |  |
| Total score | 11 | 3 |  |  |

8.Varshney

2009

| **Study objective** | Yes | Partial | unclear | No |
| --- | --- | --- | --- | --- |
| 1.Was the hypothesis/aim/objective of the study clearly stated? Study design | 1 |  |  |  |
| **Study design** |  |  |  |  |
| 2.Was the study conducted prospectively? |  |  |  | 1 |
| 3.Were the cases collected in more than one center? |  |  |  | 1 |
| 4.Were patients recruited consecutively? |  |  | 1 |  |
| **Study population** |  |  |  |  |
| 5. Were the characteristics of the patients included in the study described? |  | 1 |  |  |
| 6. Were the eligibility criteria (i.e. inclusion and exclusion criteria) for entry into the study clearly stated? |  |  |  | 1 |
| 7. Did patients enter the study at a similar point in the disease? |  |  |  | 1 |
| **Intervention and cointervention** |  |  |  |  |
| 8.Was the intervention of interest clearly described? |  | 1 |  |  |
| 9.Were additional interventions (cointerventions) clearly described? |  | 1 |  |  |
| **Outcome measures** |  |  |  |  |
| 10.Were relevant outcome measures established a priori? | 1 |  |  |  |
| 11.Were outcome assessors blinded to the intervention that patients received? |  |  | 1 |  |
| 12.Were the relevant outcomes measured using appropriate objective/subjective methods? | 1 |  |  |  |
| 13.Were the relevant outcome measures made before and after the intervention? |  |  |  | 1 |
| **Statistical analysis** |  |  |  |  |
| 14.Were the statistical tests used to assess the relevant outcomes appropriate? | 1 |  |  |  |
| **Results and conclusions** |  |  |  |  |
| 15.Was follow-up long enough for important events and outcomes to occur? | 1 |  |  |  |
| 16.Were losses to follow-up reported? | 1 |  |  |  |
| 17.Did the study provided estimates of random variability in the data analysis of relevant outcomes? |  |  | 1 |  |
| 18.Were the adverse events reported? | 1 |  |  |  |
| 19.Were the conclusions of the study supported by the results? | 1 |  |  |  |
| **Competing interests and sources of support** |  |  |  |  |
| 20.Were both competing interests and sources of support for the study reported? |  | 1 |  |  |
| Total score | 8 | 4 |  |  |

9.Dhammi

2005

| **Study objective** | Yes | Partial | unclear | No |
| --- | --- | --- | --- | --- |
| 1.Was the hypothesis/aim/objective of the study clearly stated? Study design | 1 |  |  |  |
| **Study design** |  |  |  |  |
| 2.Was the study conducted prospectively? |  |  |  | 1 |
| 3.Were the cases collected in more than one center? |  |  |  | 1 |
| 4.Were patients recruited consecutively? |  |  | 1 |  |
| **Study population** |  |  |  |  |
| 5. Were the characteristics of the patients included in the study described? |  | 1 |  |  |
| 6. Were the eligibility criteria (i.e. inclusion and exclusion criteria) for entry into the study clearly stated? |  |  |  | 1 |
| 7. Did patients enter the study at a similar point in the disease? | 1 |  |  |  |
| **Intervention and cointervention** |  |  |  |  |
| 8.Was the intervention of interest clearly described? |  | 1 |  |  |
| 9.Were additional interventions (cointerventions) clearly described? |  | 1 |  |  |
| **Outcome measures** |  |  |  |  |
| 10.Were relevant outcome measures established a priori? | 1 |  |  |  |
| 11.Were outcome assessors blinded to the intervention that patients received? |  |  | 1 |  |
| 12.Were the relevant outcomes measured using appropriate objective/subjective methods? | 1 |  |  |  |
| 13.Were the relevant outcome measures made before and after the intervention? |  |  |  | 1 |
| **Statistical analysis** |  |  |  |  |
| 14.Were the statistical tests used to assess the relevant outcomes appropriate? |  |  | 1 |  |
| **Results and conclusions** |  |  |  |  |
| 15.Was follow-up long enough for important events and outcomes to occur? | 1 |  |  |  |
| 16.Were losses to follow-up reported? | 1 |  |  |  |
| 17.Did the study provided estimates of random variability in the data analysis of relevant outcomes? |  |  | 1 |  |
| 18.Were the adverse events reported? | 1 |  |  |  |
| 19.Were the conclusions of the study supported by the results? | 1 |  |  |  |
| **Competing interests and sources of support** |  |  |  |  |
| 20.Were both competing interests and sources of support for the study reported? |  |  |  | 1 |
| Total score | 8 | 3 |  |  |

10.Emre

2005

| **Study objective** | Yes | Partial | unclear | No |
| --- | --- | --- | --- | --- |
| 1.Was the hypothesis/aim/objective of the study clearly stated? Study design | 1 |  |  |  |
| **Study design** |  |  |  | 1 |
| 2.Was the study conducted prospectively? |  |  |  | 1 |
| 3.Were the cases collected in more than one center? |  |  |  | 1 |
| 4.Were patients recruited consecutively? |  |  | 1 |  |
| **Study population** |  |  |  |  |
| 5. Were the characteristics of the patients included in the study described? |  | 1 |  |  |
| 6. Were the eligibility criteria (i.e. inclusion and exclusion criteria) for entry into the study clearly stated? |  |  |  | 1 |
| 7. Did patients enter the study at a similar point in the disease? | 1 |  |  |  |
| **Intervention and cointervention** |  |  |  |  |
| 8.Was the intervention of interest clearly described? |  | 1 |  |  |
| 9.Were additional interventions (cointerventions) clearly described? |  | 1 |  |  |
| **Outcome measures** |  |  |  |  |
| 10.Were relevant outcome measures established a priori? | 1 |  |  |  |
| 11.Were outcome assessors blinded to the intervention that patients received? |  |  | 1 |  |
| 12.Were the relevant outcomes measured using appropriate objective/subjective methods? | 1 |  |  |  |
| 13.Were the relevant outcome measures made before and after the intervention? |  |  |  |  |
| **Statistical analysis** |  |  |  | 1 |
| 14.Were the statistical tests used to assess the relevant outcomes appropriate? |  |  | 1 |  |
| **Results and conclusions** |  |  |  |  |
| 15.Was follow-up long enough for important events and outcomes to occur? | 1 |  |  |  |
| 16.Were losses to follow-up reported? |  |  |  | 1 |
| 17.Did the study provided estimates of random variability in the data analysis of relevant outcomes? |  |  | 1 |  |
| 18.Were the adverse events reported? | 1 |  |  |  |
| 19.Were the conclusions of the study supported by the results? | 1 |  |  |  |
| **Competing interests and sources of support** |  |  |  |  |
| 20.Were both competing interests and sources of support for the study reported? |  |  |  | 1 |
| Total score | 7 | 3 |  |  |

11.Flynn

2002

| **Study objective** | Yes | Partial | unclear | No |
| --- | --- | --- | --- | --- |
| 1.Was the hypothesis/aim/objective of the study clearly stated? Study design | 1 |  |  |  |
| **Study design** |  |  |  |  |
| 2.Was the study conducted prospectively? |  |  |  | 1 |
| 3.Were the cases collected in more than one center? |  |  |  | 1 |
| 4.Were patients recruited consecutively? |  |  |  | 1 |
| **Study population** |  |  |  |  |
| 5. Were the characteristics of the patients included in the study described? |  | 1 |  |  |
| 6. Were the eligibility criteria (i.e. inclusion and exclusion criteria) for entry into the study clearly stated? |  |  |  | 1 |
| 7. Did patients enter the study at a similar point in the disease? | 1 |  |  |  |
| **Intervention and cointervention** |  |  |  |  |
| 8.Was the intervention of interest clearly described? |  | 1 |  |  |
| 9.Were additional interventions (cointerventions) clearly described? |  |  |  | 1 |
| **Outcome measures** |  |  |  |  |
| 10.Were relevant outcome measures established a priori? | 1 |  |  |  |
| 11.Were outcome assessors blinded to the intervention that patients received? |  |  | 1 |  |
| 12.Were the relevant outcomes measured using appropriate objective/subjective methods? | 1 |  |  |  |
| 13.Were the relevant outcome measures made before and after the intervention? |  |  |  | 1 |
| **Statistical analysis** |  |  |  |  |
| 14.Were the statistical tests used to assess the relevant outcomes appropriate? |  |  |  | 1 |
| **Results and conclusions** |  |  |  |  |
| 15.Was follow-up long enough for important events and outcomes to occur? | 1 |  |  |  |
| 16.Were losses to follow-up reported? | 1 |  |  |  |
| 17.Did the study provided estimates of random variability in the data analysis of relevant outcomes? |  |  | 1 |  |
| 18.Were the adverse events reported? | 1 |  |  |  |
| 19.Were the conclusions of the study supported by the results? | 1 |  |  |  |
| **Competing interests and sources of support** |  |  |  |  |
| 20.Were both competing interests and sources of support for the study reported? |  | 1 |  |  |
| Total score | 8 | 3 |  |  |

12.Bagatur

2002

| **Study objective** | Yes | Partial | unclear | No |
| --- | --- | --- | --- | --- |
| 1.Was the hypothesis/aim/objective of the study clearly stated? Study design | 1 |  |  |  |
| **Study design** |  |  |  |  |
| 2.Was the study conducted prospectively? |  |  |  | 1 |
| 3.Were the cases collected in more than one center? |  |  |  | 1 |
| 4.Were patients recruited consecutively? |  |  |  | 1 |
| **Study population** |  |  |  |  |
| 5. Were the characteristics of the patients included in the study described? |  | 1 |  |  |
| 6. Were the eligibility criteria (i.e. inclusion and exclusion criteria) for entry into the study clearly stated? |  | 1 |  |  |
| 7. Did patients enter the study at a similar point in the disease? | 1 |  |  |  |
| **Intervention and cointervention** |  |  |  |  |
| 8.Was the intervention of interest clearly described? |  |  |  | 1 |
| 9.Were additional interventions (cointerventions) clearly described? |  |  |  | 1 |
| **Outcome measures** |  |  |  |  |
| 10.Were relevant outcome measures established a priori? | 1 |  |  |  |
| 11.Were outcome assessors blinded to the intervention that patients received? |  |  | 1 |  |
| 12.Were the relevant outcomes measured using appropriate objective/subjective methods? | 1 |  |  |  |
| 13.Were the relevant outcome measures made before and after the intervention? |  |  |  | 1 |
| **Statistical analysis** |  |  |  |  |
| 14.Were the statistical tests used to assess the relevant outcomes appropriate? |  |  | 1 |  |
| **Results and conclusions** |  |  |  |  |
| 15.Was follow-up long enough for important events and outcomes to occur? | 1 |  |  |  |
| 16.Were losses to follow-up reported? | 1 |  |  |  |
| 17.Did the study provided estimates of random variability in the data analysis of relevant outcomes? |  |  | 1 |  |
| 18.Were the adverse events reported? | 1 |  |  |  |
| 19.Were the conclusions of the study supported by the results? |  |  | 1 |  |
| **Competing interests and sources of support** |  |  |  |  |
| 20.Were both competing interests and sources of support for the study reported? |  |  |  | 1 |
| Total score | 7 | 2 |  |  |

13.Mirdad

2002

| **Study objective** | Yes | Partial | unclear | No |
| --- | --- | --- | --- | --- |
| 1.Was the hypothesis/aim/objective of the study clearly stated? Study design | 1 |  |  |  |
| **Study design** |  |  |  |  |
| 2.Was the study conducted prospectively? |  |  |  | 1 |
| 3.Were the cases collected in more than one center? |  |  |  | 1 |
| 4.Were patients recruited consecutively? |  |  |  | 1 |
| **Study population** |  |  |  |  |
| 5. Were the characteristics of the patients included in the study described? |  | 1 |  |  |
| 6. Were the eligibility criteria (i.e. inclusion and exclusion criteria) for entry into the study clearly stated? |  |  |  | 1 |
| 7. Did patients enter the study at a similar point in the disease? | 1 |  |  |  |
| **Intervention and cointervention** |  |  |  |  |
| 8.Was the intervention of interest clearly described? |  |  |  | 1 |
| 9.Were additional interventions (cointerventions) clearly described? |  |  |  | 1 |
| **Outcome measures** |  |  |  |  |
| 10.Were relevant outcome measures established a priori? | 1 |  |  |  |
| 11.Were outcome assessors blinded to the intervention that patients received? |  |  | 1 |  |
| 12.Were the relevant outcomes measured using appropriate objective/subjective methods? | 1 |  |  |  |
| 13.Were the relevant outcome measures made before and after the intervention? |  |  |  | 1 |
| **Statistical analysis** |  |  |  |  |
| 14.Were the statistical tests used to assess the relevant outcomes appropriate? |  |  | 1 |  |
| **Results and conclusions** |  |  |  |  |
| 15.Was follow-up long enough for important events and outcomes to occur? | 1 |  |  |  |
| 16.Were losses to follow-up reported? | 1 |  |  |  |
| 17.Did the study provided estimates of random variability in the data analysis of relevant outcomes? |  |  |  | 1 |
| 18.Were the adverse events reported? | 1 |  |  |  |
| 19.Were the conclusions of the study supported by the results? |  |  |  |  |
| **Competing interests and sources of support** | 1 |  |  |  |
| 20.Were both competing interests and sources of support for the study reported? |  |  |  | 1 |
| Total score | 8 | 1 |  |  |

14.Morsy

2001

| **Study objective** | Yes | Partial | unclear | No |
| --- | --- | --- | --- | --- |
| 1.Was the hypothesis/aim/objective of the study clearly stated? Study design | 1 |  |  |  |
| **Study design** |  |  |  |  |
| 2.Was the study conducted prospectively? |  |  |  | 1 |
| 3.Were the cases collected in more than one center? |  |  |  | 1 |
| 4.Were patients recruited consecutively? |  |  | 1 |  |
| **Study population** |  |  |  |  |
| 5. Were the characteristics of the patients included in the study described? |  | 1 |  |  |
| 6. Were the eligibility criteria (i.e. inclusion and exclusion criteria) for entry into the study clearly stated? |  |  |  | 1 |
| 7. Did patients enter the study at a similar point in the disease? | 1 |  |  |  |
| **Intervention and cointervention** |  |  |  |  |
| 8.Was the intervention of interest clearly described? |  |  |  | 1 |
| 9.Were additional interventions (cointerventions) clearly described? |  |  |  | 1 |
| **Outcome measures** |  |  |  |  |
| 10.Were relevant outcome measures established a priori? | 1 |  |  |  |
| 11.Were outcome assessors blinded to the intervention that patients received? |  |  | 1 |  |
| 12.Were the relevant outcomes measured using appropriate objective/subjective methods? | 1 |  |  |  |
| 13.Were the relevant outcome measures made before and after the intervention? |  |  |  | 1 |
| **Statistical analysis** |  |  |  |  |
| 14.Were the statistical tests used to assess the relevant outcomes appropriate? |  |  | 1 |  |
| **Results and conclusions** |  |  |  |  |
| 15.Was follow-up long enough for important events and outcomes to occur? | 1 |  |  |  |
| 16.Were losses to follow-up reported? | 1 |  |  |  |
| 17.Did the study provided estimates of random variability in the data analysis of relevant outcomes? |  |  | 1 |  |
| 18.Were the adverse events reported? | 1 |  |  |  |
| 19.Were the conclusions of the study supported by the results? | 1 |  |  |  |
| **Competing interests and sources of support** |  |  |  |  |
| 20.Were both competing interests and sources of support for the study reported? |  |  |  | 1 |
| Total score | 8 | 1 |  |  |

15.Ng, G. P

1996

| **Study objective** | Yes | Partial | unclear | No |
| --- | --- | --- | --- | --- |
| 1.Was the hypothesis/aim/objective of the study clearly stated? Study design | 1 |  |  |  |
| **Study design** |  |  |  |  |
| 2.Was the study conducted prospectively? |  |  |  | 1 |
| 3.Were the cases collected in more than one center? |  |  |  | 1 |
| 4.Were patients recruited consecutively? |  |  |  | 1 |
| **Study population** |  |  |  |  |
| 5. Were the characteristics of the patients included in the study described? |  | 1 |  |  |
| 6. Were the eligibility criteria (i.e. inclusion and exclusion criteria) for entry into the study clearly stated? |  | 1 |  |  |
| 7. Did patients enter the study at a similar point in the disease? | 1 |  |  |  |
| **Intervention and cointervention** |  |  |  |  |
| 8.Was the intervention of interest clearly described? |  | 1 |  |  |
| 9.Were additional interventions (cointerventions) clearly described? |  | 1 |  |  |
| **Outcome measures** |  |  |  |  |
| 10.Were relevant outcome measures established a priori? |  |  |  | 1 |
| 11.Were outcome assessors blinded to the intervention that patients received? |  |  | 1 |  |
| 12.Were the relevant outcomes measured using appropriate objective/subjective methods? | 1 |  |  |  |
| 13.Were the relevant outcome measures made before and after the intervention? |  |  |  | 1 |
| **Statistical analysis** |  |  |  |  |
| 14.Were the statistical tests used to assess the relevant outcomes appropriate? | 1 |  |  |  |
| **Results and conclusions** |  |  |  |  |
| 15.Was follow-up long enough for important events and outcomes to occur? | 1 |  |  |  |
| 16.Were losses to follow-up reported? | 1 |  |  |  |
| 17.Did the study provided estimates of random variability in the data analysis of relevant outcomes? |  |  | 1 |  |
| 18.Were the adverse events reported? | 1 |  |  |  |
| 19.Were the conclusions of the study supported by the results? | 1 |  |  |  |
| **Competing interests and sources of support** |  |  |  |  |
| 20.Were both competing interests and sources of support for the study reported? |  |  |  | 1 |
| Total score | 8 | 4 |  |  |

16.Forlin

1992

| **Study objective** | Yes | Partial | unclear | No |
| --- | --- | --- | --- | --- |
| 1.Was the hypothesis/aim/objective of the study clearly stated? Study design | 1 |  |  |  |
| **Study design** |  |  |  |  |
| 2.Was the study conducted prospectively? |  |  |  | 1 |
| 3.Were the cases collected in more than one center? |  |  |  | 1 |
| 4.Were patients recruited consecutively? |  |  |  | 1 |
| **Study population** |  |  |  |  |
| 5. Were the characteristics of the patients included in the study described? |  | 1 |  |  |
| 6. Were the eligibility criteria (i.e. inclusion and exclusion criteria) for entry into the study clearly stated? | 1 |  |  |  |
| 7. Did patients enter the study at a similar point in the disease? | 1 |  |  |  |
| **Intervention and cointervention** |  |  |  |  |
| 8.Was the intervention of interest clearly described? |  |  |  | 1 |
| 9.Were additional interventions (cointerventions) clearly described? |  |  |  | 1 |
| **Outcome measures** |  |  |  |  |
| 10.Were relevant outcome measures established a priori? |  |  |  | 1 |
| 11.Were outcome assessors blinded to the intervention that patients received? |  |  | 1 |  |
| 12.Were the relevant outcomes measured using appropriate objective/subjective methods? | 1 |  |  |  |
| 13.Were the relevant outcome measures made before and after the intervention? |  |  |  | 1 |
| **Statistical analysis** |  |  |  |  |
| 14.Were the statistical tests used to assess the relevant outcomes appropriate? |  |  |  | 1 |
| **Results and conclusions** |  |  |  |  |
| 15.Was follow-up long enough for important events and outcomes to occur? | 1 |  |  |  |
| 16.Were losses to follow-up reported? | 1 |  |  |  |
| 17.Did the study provided estimates of random variability in the data analysis of relevant outcomes? |  |  | 1 |  |
| 18.Were the adverse events reported? | 1 |  |  |  |
| 19.Were the conclusions of the study supported by the results? | 1 |  |  |  |
| **Competing interests and sources of support** |  |  |  |  |
| 20.Were both competing interests and sources of support for the study reported? |  |  |  | 1 |
| Total score | 8 | 1 |  |  |

| **Study objective** | Yes | Partial | unclear | No |
| --- | --- | --- | --- | --- |
| 1.Was the hypothesis/aim/objective of the study clearly stated? Study design | 1 |  |  |  |
| **Study design** |  |  |  |  |
| 2.Was the study conducted prospectively? |  |  |  | 1 |
| 3.Were the cases collected in more than one center? |  |  |  | 1 |
| 4.Were patients recruited consecutively? |  |  | 1 |  |
| **Study population** |  |  |  |  |
| 5. Were the characteristics of the patients included in the study described? |  | 1 |  |  |
| 6. Were the eligibility criteria (i.e. inclusion and exclusion criteria) for entry into the study clearly stated? |  | 1 |  |  |
| 7. Did patients enter the study at a similar point in the disease? | 1 |  |  |  |
| **Intervention and cointervention** |  |  |  |  |
| 8.Was the intervention of interest clearly described? |  |  |  | 1 |
| 9.Were additional interventions (cointerventions) clearly described? |  |  |  | 1 |
| **Outcome measures** |  |  |  |  |
| 10.Were relevant outcome measures established a priori? |  |  |  | 1 |
| 11.Were outcome assessors blinded to the intervention that patients received? |  |  | 1 |  |
| 12.Were the relevant outcomes measured using appropriate objective/subjective methods? | 1 |  |  |  |
| 13.Were the relevant outcome measures made before and after the intervention? |  |  |  | 1 |
| **Statistical analysis** |  |  |  |  |
| 14.Were the statistical tests used to assess the relevant outcomes appropriate? |  |  |  | 1 |
| **Results and conclusions** |  |  |  |  |
| 15.Was follow-up long enough for important events and outcomes to occur? | 1 |  |  |  |
| 16.Were losses to follow-up reported? | 1 |  |  |  |
| 17.Did the study provided estimates of random variability in the data analysis of relevant outcomes? |  |  | 1 |  |
| 18.Were the adverse events reported? | 1 |  |  |  |
| 19.Were the conclusions of the study supported by the results? |  |  | 1 |  |
| **Competing interests and sources of support** |  |  |  |  |
| 20.Were both competing interests and sources of support for the study reported? |  |  |  | 1 |
| Total score | 6 | 2 |  |  |

17.Canale

1977

18.Chong

1975

| **Study objective** | Yes | Partial | unclear | No |
| --- | --- | --- | --- | --- |
| 1.Was the hypothesis/aim/objective of the study clearly stated? Study design | 1 |  |  |  |
| **Study design** |  |  |  |  |
| 2.Was the study conducted prospectively? |  |  |  | 1 |
| 3.Were the cases collected in more than one center? |  |  |  | 1 |
| 4.Were patients recruited consecutively? |  |  |  | 1 |
| **Study population** |  |  |  |  |
| 5. Were the characteristics of the patients included in the study described? |  | 1 |  |  |
| 6. Were the eligibility criteria (i.e. inclusion and exclusion criteria) for entry into the study clearly stated? |  | 1 |  |  |
| 7. Did patients enter the study at a similar point in the disease? | 1 |  |  |  |
| **Intervention and cointervention** |  |  |  |  |
| 8.Was the intervention of interest clearly described? |  |  |  | 1 |
| 9.Were additional interventions (cointerventions) clearly described? |  |  |  | 1 |
| **Outcome measures** |  |  |  |  |
| 10.Were relevant outcome measures established a priori? |  |  |  | 1 |
| 11.Were outcome assessors blinded to the intervention that patients received? |  |  | 1 |  |
| 12.Were the relevant outcomes measured using appropriate objective/subjective methods? | 1 |  |  |  |
| 13.Were the relevant outcome measures made before and after the intervention? |  |  |  | 1 |
| **Statistical analysis** |  |  |  |  |
| 14.Were the statistical tests used to assess the relevant outcomes appropriate? |  |  |  | 1 |
| **Results and conclusions** |  |  |  |  |
| 15.Was follow-up long enough for important events and outcomes to occur? | 1 |  |  |  |
| 16.Were losses to follow-up reported? | 1 |  |  |  |
| 17.Did the study provided estimates of random variability in the data analysis of relevant outcomes? |  |  | 1 |  |
| 18.Were the adverse events reported? | 1 |  |  |  |
| 19.Were the conclusions of the study supported by the results? | 1 |  |  |  |
| **Competing interests and sources of support** |  |  |  |  |
| 20.Were both competing interests and sources of support for the study reported? |  |  |  | 1 |
| Total score | 7 | 2 |  |  |

19.Zolczer

1972

| **Study objective** | Yes | Partial | unclear | No |
| --- | --- | --- | --- | --- |
| 1.Was the hypothesis/aim/objective of the study clearly stated? Study design | 1 |  |  |  |
| **Study design** |  |  |  |  |
| 2.Was the study conducted prospectively? |  |  |  | 1 |
| 3.Were the cases collected in more than one center? |  |  |  | 1 |
| 4.Were patients recruited consecutively? |  |  |  | 1 |
| **Study population** |  |  |  |  |
| 5. Were the characteristics of the patients included in the study described? |  | 1 |  |  |
| 6. Were the eligibility criteria (i.e. inclusion and exclusion criteria) for entry into the study clearly stated? |  |  |  | 1 |
| 7. Did patients enter the study at a similar point in the disease? | 1 |  |  |  |
| **Intervention and cointervention** |  |  |  |  |
| 8.Was the intervention of interest clearly described? |  |  |  | 1 |
| 9.Were additional interventions (cointerventions) clearly described? |  |  |  | 1 |
| **Outcome measures** |  |  |  |  |
| 10.Were relevant outcome measures established a priori? |  |  |  | 1 |
| 11.Were outcome assessors blinded to the intervention that patients received? |  |  | 1 |  |
| 12.Were the relevant outcomes measured using appropriate objective/subjective methods? | 1 |  |  |  |
| 13.Were the relevant outcome measures made before and after the intervention? |  |  |  | 1 |
| **Statistical analysis** |  |  |  |  |
| 14.Were the statistical tests used to assess the relevant outcomes appropriate? |  |  |  | 1 |
| **Results and conclusions** |  |  |  |  |
| 15.Was follow-up long enough for important events and outcomes to occur? | 1 |  |  |  |
| 16.Were losses to follow-up reported? | 1 |  |  |  |
| 17.Did the study provided estimates of random variability in the data analysis of relevant outcomes? |  |  | 1 |  |
| 18.Were the adverse events reported? | 1 |  |  |  |
| 19.Were the conclusions of the study supported by the results? | 1 |  |  |  |
| **Competing interests and sources of support** |  |  |  |  |
| 20.Were both competing interests and sources of support for the study reported? |  |  |  | 1 |
| Total score | 7 | 1 |  |  |

| **Study objective** | Yes | Partial | unclear | No |
| --- | --- | --- | --- | --- |
| 1.Was the hypothesis/aim/objective of the study clearly stated? Study design | 1 |  |  |  |
| **Study design** |  |  |  |  |
| 2.Was the study conducted prospectively? |  |  |  | 1 |
| 3.Were the cases collected in more than one center? |  |  |  | 1 |
| 4.Were patients recruited consecutively? |  |  |  | 1 |
| **Study population** |  |  |  |  |
| 5. Were the characteristics of the patients included in the study described? |  | 1 |  |  |
| 6. Were the eligibility criteria (i.e. inclusion and exclusion criteria) for entry into the study clearly stated? |  |  |  | 1 |
| 7. Did patients enter the study at a similar point in the disease? | 1 |  |  |  |
| **Intervention and cointervention** |  |  |  |  |
| 8.Was the intervention of interest clearly described? |  | 1 |  |  |
| 9.Were additional interventions (cointerventions) clearly described? |  |  |  | 1 |
| **Outcome measures** |  |  |  |  |
| 10.Were relevant outcome measures established a priori? | 1 |  |  |  |
| 11.Were outcome assessors blinded to the intervention that patients received? |  |  | 1 |  |
| 12.Were the relevant outcomes measured using appropriate objective/subjective methods? | 1 |  |  |  |
| 13.Were the relevant outcome measures made before and after the intervention? |  |  |  | 1 |
| **Statistical analysis** |  |  |  |  |
| 14.Were the statistical tests used to assess the relevant outcomes appropriate? |  |  |  | 1 |
| **Results and conclusions** |  |  |  |  |
| 15.Was follow-up long enough for important events and outcomes to occur? | 1 |  |  |  |
| 16.Were losses to follow-up reported? | 1 |  |  |  |
| 17.Did the study provided estimates of random variability in the data analysis of relevant outcomes? |  |  | 1 |  |
| 18.Were the adverse events reported? | 1 |  |  |  |
| 19.Were the conclusions of the study supported by the results? | 1 |  |  |  |
| **Competing interests and sources of support** |  |  |  |  |
| 20.Were both competing interests and sources of support for the study reported? |  |  |  | 1 |
| Total score | 8 | 2 |  |  |

20.Lam

1971

21.Ratliff

1962

| **Study objective** | Yes | Partial | unclear | No |
| --- | --- | --- | --- | --- |
| 1.Was the hypothesis/aim/objective of the study clearly stated? Study design | 1 |  |  |  |
| **Study design** |  |  |  |  |
| 2.Was the study conducted prospectively? |  |  |  | 1 |
| 3.Were the cases collected in more than one center? |  |  |  | 1 |
| 4.Were patients recruited consecutively? |  |  |  | 1 |
| **Study population** |  |  |  |  |
| 5. Were the characteristics of the patients included in the study described? |  | 1 |  |  |
| 6. Were the eligibility criteria (i.e. inclusion and exclusion criteria) for entry into the study clearly stated? |  |  |  | 1 |
| 7. Did patients enter the study at a similar point in the disease? |  |  |  | 1 |
| **Intervention and cointervention** |  |  |  |  |
| 8.Was the intervention of interest clearly described? |  | 1 |  |  |
| 9.Were additional interventions (cointerventions) clearly described? |  | 1 |  |  |
| **Outcome measures** |  |  |  |  |
| 10.Were relevant outcome measures established a priori? |  |  |  | 1 |
| 11.Were outcome assessors blinded to the intervention that patients received? |  |  | 1 |  |
| 12.Were the relevant outcomes measured using appropriate objective/subjective methods? | 1 |  |  |  |
| 13.Were the relevant outcome measures made before and after the intervention? |  |  |  | 1 |
| **Statistical analysis** |  |  |  |  |
| 14.Were the statistical tests used to assess the relevant outcomes appropriate? |  |  | 1 |  |
| **Results and conclusions** |  |  |  |  |
| 15.Was follow-up long enough for important events and outcomes to occur? | 1 |  |  |  |
| 16.Were losses to follow-up reported? | 1 |  |  |  |
| 17.Did the study provided estimates of random variability in the data analysis of relevant outcomes? |  |  | 1 |  |
| 18.Were the adverse events reported? | 1 |  |  |  |
| 19.Were the conclusions of the study supported by the results? | 1 |  |  |  |
| **Competing interests and sources of support** |  |  |  |  |
| 20.Were both competing interests and sources of support for the study reported? |  |  |  | 1 |
| Total score | 6 | 3 |  |  |

More than15: 0 15-11: 9 6-10: 12 lower 5: 0
